# Synthetic reprogramming of tumor cell fate for modulating radiotherapy-induced dynamic responses: perspectives on radiosensitizing and immunoregulatory effects

**DOI:** 10.7150/thno.119822

**Published:** 2025-08-30

**Authors:** Wei Zhou, Lulu Wang, Hui Zhang, Yongzhong Wu, Menghuan Li, Zhong Luo

**Affiliations:** 1 Radiotherapy Center, Chongqing University Cancer Hospital, Chongqing, China.; 2 Department of Breast, Chongqing Hospital of Traditional Chinese Medicine, Chongqing City, China.; 3 School of Life Science, Chongqing University, Chongqing, China.

**Keywords:** Cell fate determination, radiosensitization, immunoregulation, synthetic therapeutics.

## Abstract

Radiotherapy is one of the most commonly prescribed anticancer modalities in the clinic, which is widely recognized as an effective and safe treatment for a broad spectrum of solid tumor indications. Interestingly, there is increasing evidence that the tumors can dynamically modulate cell fate decisions after ionizing radiation (IR) exposure, which is beneficial for escaping the radiation-induced antitumorigenic cell damaging and immunostimulatory impacts. Consequently, the regulatory network of cell fate determination could be a promising target for enhancing the susceptibility of tumor cells to various radiotherapeutic modalities. In this review, we provide a comprehensive account on the mechanisms of post-radiation cell fate control in tumor cells to escape programmed cell death (PCD) including apoptosis, necrosis, pyroptosis and ferroptosis, while special emphasis is placed on the development of synthetic agents for the therapeutic modulation of post-radiation tumor cell fate decisions to facilitate tumor cell eradication, focusing on their therapeutic utility for amplifying the RT-induced direct tumor cell damage as well as promoting the post-IR antitumor immunity. We envision that these synthetic cell fate regulatory technologies could provide new avenues for improving radiotherapeutic efficacy.

## Introduction

Malignant tumors are major threats to human health and well-being worldwide that continue to demand innovations in cancer therapeutics 1, 2. Radiotherapy is one of the most widely used antitumor modalities that employs high-energy IR such as photon and particle beams to damage tumor cells and tissues, allowing precise ablation of tumors in localized areas with reduced invasiveness and systemic toxicity 3, 4. Owing to these merits, radiotherapy is commonly prescribed as the first-line treatment for a broad spectrum of solid tumor indications. However, the actual efficacy of radiotherapy in the clinic is still not satisfactory with insufficient tumor cell inhibition, high relapse rate and unneglectable radiation-associated adverse events 5. Indeed, effective radiosensitization has been one of the primary goals in clinical oncology in recent decades. Most of the research in this area focuses on the implementation of high Z metal species as sensitizing agents, which have much higher radiation absorption rate than the low-density soft tissues 6, 7. Owing to the high atomic number, high Z metal species could more efficiently absorb X-ray photons through photoelectric interaction to emit secondary electrons including photoelectrons and Auger electrons, thus establishing a highly intensified radiation dose in vicinity of the metal species. On the other hand, the metal species exert catalyst-like function to promote the radiolysis of proximal water molecules for generating abundant reactive oxygen species (ROS). The high Z metal-enabled radiosensitization effect could substantially enhance the post-RT damage to biomolecules including DNA, proteins and lipids for initiating various cell death programs. Based on a balanced consideration of radiosensitizing efficacy and *in vivo* safety, the most extensively explored high Z metal species in the clinic include gold, platinum, gadolinium, hafnium, etc. Alternative to enhancing the direct RT-tumor interactions, there is increasing interest to abolish the intrinsic resistance mechanisms in tumor cells to enhance their susceptibility to RT-induced antitumor effects by analyzing the tumor-associated adaptive cellular programs after radiation exposure, which may yield new radiosensitizing strategies to improve radiotherapeutic efficacy.

It is well established that tumorigenesis is intrinsically linked to aberrations in cell fate determination, based on which tumor cells are capable of evading PCD and proliferate in an unrestrained manner 8, 9. Tumor cells are under constant survival stresses such as acidic microenvironment, hypoxia, nutrient deprivation and genetic instability, all of which are potent apoptosis stimuli 10-12. A universal trait of tumor cells is that they are capable of disabling apoptosis signaling pathways by modulating the expression and functions of key apoptosis mediators including B cell lymphoma 2 (BCL-2), BCL2-Associated X (BAX), retinoblastoma protein 1, caspases, etc 13, 14. Meanwhile, tumor cells universally demonstrate impaired neurofibromatosis type 2 and liver kinase B1 activities as well as elevated activation of Hippo signaling, which may abolish contact inhibition while promoting tumor progression 15. Tumor cells can also genetically deactivate p53 and pRb while reactivating telomerase to evade senescence and achieve replicative immortality 16. Notably, recent insights increasingly demonstrate that the aberrant cell fate control in tumor cells is also crucial for resisting survival stresses induced by various cytotoxic treatments such as chemotherapeutics and radiotherapy 17, 18. Particularly, tumor cells are capable of leveraging multiple cell fate decisions after radiation exposure to evade radiation-induced PCD and attenuate post-radiation immune responses, and some of the most important mechanisms include apoptosis, necrosis, pyroptosis, ferroptosis, autophagy and senescence. Consequently, the cancer-intrinsic cell fate regulatory network has emerged as a promising target for radiosensitization of various solid tumor indications, but the therapeutic development in this field is severely impeded by the lack of clinically applicable molecular mediators. For instance, cell fate regulators such as Kirsten rat sarcoma viral oncogene homolog (KRAS) and p53 are well-known non-druggable targets that still have no clinically approved inhibitors 19, 20. Therapeutic modulation of post-radiation tumor cell fate determination thus remains a promising yet underexplored option for tumor radiosensitization.

Interestingly, the recent advances in synthetic biotechnology offer novel opportunities for addressing the insufficiencies in tumor cell fate decision regulation, holding immense potential to overcome the tumor-intrinsic radioresistance. In this review, we first desiccate the cell fate determination mechanisms in tumor cells that govern their pro-survival responses after radiotherapy including apoptosis, necrosis, ferroptosis, pyroptosis, autophagy and senescence. We then summarize the current progress in the development of synthetic agents for the therapeutic modulation of post-radiation tumor cell fate decisions to amplify the antitumor efficacy by (1) enhancing the RT-induced lethal effects and (2) promoting post-IR antitumor immune responses. A perspective is also included discussing the challenges and potential breakthroughs in cell fate regulatory synthetic drugs for tumor radiosensitization from a translational perspective. We envision that this review could provide new insights into tumorigenesis and progression events while facilitating the development of new auxiliary treatment modalities for enhancing radiotherapeutic efficacy in the clinic.

## Dynamic cell fate control facilitates tumor cell survival after radiation exposure

**Figure 1:**
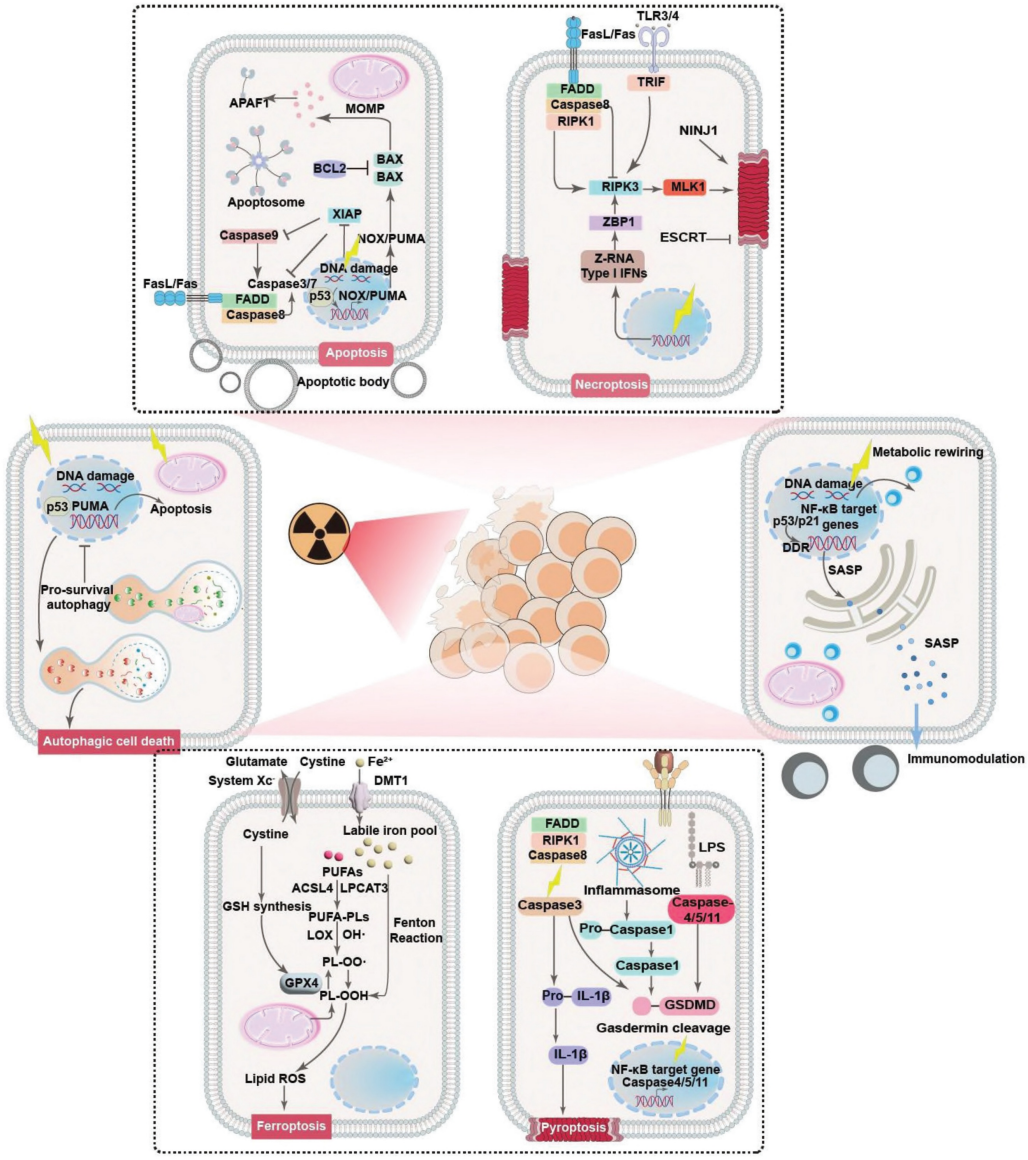
Principle mechanisms of post-radiotherapy tumor cell fate regulation and the associated molecular pathways. Radiotherapy could act as a multifaceted trigger that drives tumor cell fate towards different decisions including apoptosis, necrosis/necroptosis, pyroptosis, ferroptosis, autophagy and senescence through distinct pathways, leading to significant changes in the eventual treatment outcome through leveraging tumor cell survival and tumor-immune cell interactions.

Clinical evidence collectively demonstrates that RT rarely causes immediate tumor cell death. Instead, radiation exposure would damage key molecular and cellular components in tumor cells that prime them for PCD in the following period 21, 22. Indeed, it is widely acknowledged that radiotherapy could profoundly modify both the tumor cells and tumor microenvironment (TME), acting as a critical controller of tumor cell behaviors for driving them toward distinct cell fate decisions. The primary mechanism by which RT impacts tumor cells is through inducing various forms of DNA damage, especially DNA double-strand breaks. The initial RT-induced DNA damage would subsequently activate the DNA damage response (DDR) system to initiate the DNA repair program and cell cycle machineries as a pro-survival attempt. In the context of irreparable DNA damage of defective DDR systems, the ataxia-telangiectasia mutated-p53 axis would be activated to initiate the apoptosis cascades. Alternatively, the RT-induced cellular damage may also activate the p53-p21 axis and direct tumor cells towards a cytostatic state termed senescence. Beyond these pathways, RT may also induce high levels of ROS that eventually overwhelm the tumor-intrinsic antioxidant system, further inducing iron-dependent ferroptosis through promoting lipid peroxidation or triggering inflammatory cell death pathways like necroptosis and pyroptosis. The choice of cell fate is highly context-dependent, influenced by radiation dose/fractionation, tumor cell types, metabolic landscape and the specific genetic makeup. To survive the RT-induced cytotoxic effects and repopulate tumors, tumor cells can dynamically regulate their post-radiation cell fate decisions to enter a radiation-persistent state, thus avoiding the activation of anti-tumorigenic signaling pathways (Figure 1). In this section, a concise yet comprehensive analysis is provided regarding radiation-enforced cell fate decisions and pro-survival adaptive mechanisms in tumor cells (Table 1).

### Evasion mechanisms of post-IR PCD

The tumor inhibition effect of radiotherapy is essentially dependent on the capability of RT to drive tumor cells towards different fates, which are closely linked to the RT-intrinsic cell damage mechanisms. For instance, RT is known to induce pronounced damage to intracellular biomolecules and microstructures as well as perturbate the extracellular compartment, leading to the activation of various intrinsic and extrinsic apoptosis pathways 23. Meanwhile, the RT-induced DNA damage and cellular dyshomeostasis would lead to the activation of various necrosis-associated pathways such as cyclophilin D, mixed lineage kinase domain-like pseudokinase, receptor-interacting protein kinase family to promote cancer cell necrosis 24. Radiation exposure can also activate the Acyl-CoA Synthetase Long Chain Family Member 4 (ACSL4) expression in cancer cells to promote the biosynthesis of polyunsaturated fatty acid (PUFA)-containing phospholipids, which are highly susceptible to oxidation and may thus cooperate with the RT-induced ROS stress to trigger lipid peroxidation in cancer cells in a DNA-independent manner, eventually activating the ferroptosis cascades 25, 26. Recent studies also reveal that RT can induce inflammasome formation in cancer cells and activate multiple members in the caspase family including caspase 1, caspase 3, caspase 9, etc. to mount pyroptosis 27. Notably, there is increasing evidence showing that cancer cells can dynamically regulate their cell fate decisions to resist the acute and chronic antitumorigenic impact of IR, providing a strong rationale for promoting the sublethal effects of radiotherapy against various tumor indications.

It is widely accepted that RT-induced apoptosis forms the cornerstone of its antitumor effect, which is directly resulted from the RT-induced cellular stresses including DNA damage, elevated ROS level and death receptor signaling. Nevertheless, tumor cells frequently demonstrated intrinsic apoptosis resistance that constitutes a formidable barrier impairing the anticancer efficacy of radiotherapy. In addition, cancer cells can further alter their cell fate decisions upon radiation exposure to repair radiotherapy-induced cellular damage. Notably, the RT-induced DNA aberrations such as double-stand breaks, single-strand breaks, base/sugar modifications, adducts and crosslinking would initiate a cascade of DNA damage responses 28, 29, which include the activation of those typical DNA repair systems such as homologous recombination or nonhomologous end-joining while also inducing cell cycle arrest at G1-S and G2-M checkpoints 30, 31. Interestingly, the post-IR cell cycle arrest not only provide more time for restoring the damaged DNA, but also position the tumor cells into a duplication-ready state, which are conducive for improving the DNA repairing efficacy 32. This is primarily achieved through inhibiting cyclin-cyclin-dependent kinase (CDK) complexes, which are the primary drivers of cell cycle progression under normal circumstances 33. For instance, RT is capable of stabilizing p53 in cancer cells to transcriptionally activate CDK inhibitor p21, which further inhibits CDK2 and its complexation with cyclin A and E to elicit G1-S arrest. Alternatively, under certain circumstances where the p53-mediated G1-S checkpoint is disrupted, RT-treated tumor cells frequently demonstrate inhibited cyclin B activity that evokes G2-M arrest. The post-IR tumor cell cycle arrest is a determinant factor that tips DNA-damaged tumor cells towards different fates after radiation exposure, where the tumor cells either survival radiotherapy through adequate DNA repair or undergo apoptosis in the context of failed DNA restoration. In addition to cell cycle arrest, there is concrete evidence that RT would induce the universal activation of numerous anti-apoptosis signaling pathways in tumor cells to evade apoptosis. For instance, Jeon *et al.* discovered that RT induced the robust upregulation of Tissue factor 3 in glioblastoma cells to activate canonical phosphoinositide 3-kinase and nuclear Factor kappa B (NF-κB) signaling, both of which are pro-survival pathways capable of promoting the expression of anti-apoptotic BCL-2 proteins while inhibiting pro-apoptotic BAX proteins, thus enhancing the radioresistance of glioblastoma cells through alleviating post-IR apoptosis 34. Yang *et al.* reported that RT substantially upregulated RAS-mitogen-activated protein kinase (MAPK) signaling in tumor cells bearing KRAS mutation, thus triggering the dissociation of Nuclear factor erythroid 2-related factor 2 (NRF2) from Kelch-like ECH-associated protein 1 (KEAP1) as well as facilitating NRF2 nucleus translocation, eventually enhancing the cellular antioxidant responses to attenuate post-IR apoptosis 35. In addition, it is important to note that the tumor heterogeneity may significantly influence the tumor cell response to pro-apoptosis stimuli for promoting post-RT survival. Indeed, tumor cells may present distinct genetic mutation and expressions of key apoptosis mediators and effectors even at an intra-tumor level, leading to significant difference in their susceptibility to RT-induced pro-apoptosis cues. For instance, tumor subtypes with intrinsically higher BCL-2 expression levels are generally more resistant to RT-induced apoptosis than those BCL-2-low subtypes 36. Alternatively, tumor cells with elevated expression levels of DNA repair mediators generally have lower propensity towards apoptosis after radiotherapy 37. These observations collectively confirm that the molecular heterogeneity of tumor cells could contribute to their resistance to RT-induced apoptosis and limit the treatment responses.

Necrosis/necroptosis are also major forms of cell death involved in RT-induced tumor inhibition effect, which is the combined results of overwhelming DNA damage under high RT doses and local ischemia due to RT-induced microvessel collapse 38-44. Clinical insights collective demonstrate that tumor cells can regulate the activity of certain cell fate modifiers to evade or resist post-IR necrosis. Typically, adenosine deaminase acting on RNA 1 (ADAR1), an evolutionarily conserved RNA editing enzyme, prevents the production of Z-DNA molecules upon radiation exposure by converting adenosine residues to inosine through the interaction with the Zα domains, which would thus repress the activation of Z-DNA-binding protein 1 (ZBP1) to inhibit the receptor-interacting protein kinase 3 (RIPK3)-dependent necroptosis pathways 45-47. Alternatively, tumor cells are prone to undergoing nucleus accumulation of caspase 8 as a pro-survival response to radiotherapy, which could interact with FLICE-like inhibitory protein long (FLIP_L_) to form a proteolytic complex for inhibiting RIPK3 dependent necrotic pathways 48, 49. Nevertheless, RT mostly induces mixed apoptosis and necrosis of tumor cells due to the shared initiation and execution mechanisms such as DNA damage responses and caspase activation, and their contribution to the eventual tumor inhibition efficiency is highly dependent on the total RT dose. Specifically, low RT doses mostly induce tumor cell apoptosis, while moderate or high RT doses (>30 Gy) tend to activate the necrosis cascades 50. Consequently, it is important to take the dosage and fractionation of the radiotherapy into account when designing radiosensitization strategies by controlling post-IR cell fate decisions. Furthermore, the necroptosis sensitivity of tumor cells may also be profoundly affected by the molecular heterogeneity thereof. There is concrete evidence that the expression levels of key necroptosis mediators such as RIPK1 and RIPK3 may vary significantly among different tumor cell clones in the same tumors 51, 52, suggesting the variation of necroptosis susceptibility in heterogenous tumor cell populations may cause significant alteration in the RT responses.

The RT-induced DNA damage can potently activate various stress sensing cascades to initiate pyroptosis, which is an inflammatory cell death mode characterized by marked inflammasome activation, gasdermin-dependent plasma membrane pore formation and osmotic lysis 53-55. From a general perspective, RT can substantially enhance the ROS stress in tumor cells that readily dissociates thioredoxin-interacting protein (TXNIP) from oxidized thioredoxin-1, and the detached TXNIP will further bind to NLR family pyrin domain containing 3 (NLRP3) to trigger inflammasome formation, which is a multimolecular protein complex capable of recruiting caspase 1 and activating its pro-pyroptosis function through autocatalysis 56. The activated caspase 1 will cleave gasdermin proteins to release their N-terminal domains, which could be inserted into cell membranes and self-assemble with various phospholipids to generate membrane-puncturing ring-shaped oligomers and trigger cell death 57, 58. In addition to the classic caspase-1 dependent pathway, there are reports that RT can also activate non-classical pyroptosis pathways associated with other caspase family members including caspase-3 and caspase-9 to mediate the release of gasdermin N terminals 59. In response to treatment-induced pyroptosis stress, tumor cells harness several cell fate control mechanisms to evade cell death. Typically, El-Kenawi *et al.* reported that tumor cells may undergo enhanced methionine flux and taurine production to resist pyroptosis-associated osmotic crisis 60. The elevated utilization of methionine and taurine could not only act as organic osmolytes to maintain cell membrane integrity in the context of pyroptotic osmotic lysis to ensure tumor cell survival in the short term, but also induce metabolic reprogramming of tumor cells to elicit genome-wide hypermethylation of tumor DNA to dampen danger signal sensing systems such as metal ion transporters, suppressor of mothers against decapentaplegic (SMAD) cascades and interferon-I (IFN-I) signaling while promoting cell proliferation, thus enhancing the pyroptotic persistence of tumor cells in the long term. Su *et al.* reported that the excessive upregulation of β5-integrin in solid tumors could activate Src-STAT3 signaling to promote *N*-acylsphingosine amidohydrolase 2 (ASAH2) activity, which would reduce the cellular abundance of its metabolite ceramide and sequentially alleviate ROS stress in tumor cells through regulating mitochondrial metabolism, thus protecting tumor cells from treatment-induced pyroptosis by blocking the NLRP3-caspase 1 axis. Meanwhile, inhibiting tumor-intrinsic β5-integrin and ASAH2 activities drastically enhanced their susceptibility to gemcitabine and fluorouracil-induced canonical pyroptosis 61. These data collectively suggest that pyroptosis has an active role in the tumor inhibition activity of radiotherapy and that modulating the pyroptosis-associated cell fate determination pathways could potentially enhance the radiotherapeutic efficacy. Notably, although pyroptosis and apoptosis could both initiated by RT and share common effectors such as Caspase 3, the present distinct dosage-dependent activation threshold that may lead to significant variations in the dominant cell death forms after RT. Similar to necroptosis, the tumor cell propensity towards pyroptosis under low RT doses (below 5 Gy) is generally very low, which is due to the sublethal DNA damage thereof. Contrastingly, in the range of relatively higher RT doses (> 8 Gy), the extensive DNA damage would stimulate various stress sensors to activate inflammatory caspases, eventually switching tumor cell fate from apoptosis to pyroptosis 62. Interestingly, it is also reported that the expression levels of the pyroptosis effector gasdermin proteins may differ significantly across various cancer indications and intratumoral subtypes 63, where gasdermin-low cancer cell types tend to show enhanced resistance to RT and its pyroptosis-inducing effects 64, 65.

The RT-associated radiolysis effect would generate abundant ROS in tumor cells, which would substantially impair the redox homeostasis while promoting the iron-catalyzed peroxidation of membrane lipids, thus compromising the integrity and functions of tumor cell membranes 66-68. Considering the intrinsic connection between ferroptosis and redox dyshomeostasis, recent studies increasingly reveal that ferroptosis is involved in the radiotherapy-evoked antitumor actions. Generally speaking, ferroptosis is regulated by the interplay of iron metabolism, lipid metabolism and redox balance 69, 70. In the context of critical failures in the lipid antioxidant systems including glutathione peroxidase 4/glutathione, ferroptosis suppressor protein 1/coenzyme Q10, dihydroorotate dehydrogenase/ubiquinol, GTP cyclohydrolase 1/tetrahydrobiopterin, etc, lipid peroxides generated through the accidental ROS attack on polyunsaturated fatty acid (PUFA)-containing phospholipids cannot be detoxified timely and further undergo a complex chain reaction that results in the peroxidation of nearby phospholipids, and this process could be drastically accelerated in the presence of catalytically active iron species on account of its capacity of promoting radical formation and propagation 71. Current insights collectively demonstrate the RT could induce tumor cell ferroptosis through multiple pathways. Typically, RT could substantially elevate the ROS stress in tumor cells that could attack PUFAs in tumor cells and subtract their electrons to form PUFA radicals, which are prone to peroxidation by reacting with ambient oxygen molecules to generate ferroptosis-initiating lipid peroxyl radicals (PUFA-OO•). In addition to these direct oxidative effects, RT would also stimulate the acyl-CoA synthase long-chain 4 (ACSL4) and lysophosphatidylcholine acyltransferase 3 (LPCAT3) mediated PUFA-containing phospholipid biosynthesis and contribute to the initiation and amplification of ferroptosis. Tumor cells are known to mobilize multiple cell fate regulatory systems to escape post-IR ferroptosis. For instance, tumor cells frequently presented upregulated expression of cystine-glutamate antiporter solute carrier family 7 member 11 (SLC7A11), which may support glutathione (GSH) biosynthesis and enhance the detoxification capacity of glutathione peroxidase 4 (GPX4)/GSH system to convert cytotoxic lipid peroxides into non-toxic lipid alcohols, thus contributing to the maintenance of cellular redox homeostasis in tumor cells for blocking post-IR ferroptosis 72, 73. Tirinato *et al.* reported that X ray exposure induced a significant increase of lipid droplet (LD) contents in a broad spectrum of tumor cells through perturbating cellular iron balance. LDs are lipid-storing cellular organelles capable of sequestering ferroptosis-susceptible PUFAs from various lipid membrane structures through lipid exchange or displacement, and the post-IR LD upregulation helps to reduce the lipid peroxidation in vital cellular membranes for maintaining their structural and functional integrity 74. In addition, tumor cells are known to activate multiple cell fate mediators including p53 and adenosine monophosphate-activated protein kinase (AMPK) to rebalance iron and lipid metabolism and boost antioxidative capacity by (1) reducing PUFA-PL biosynthesis, (2) alleviating iron overload and (3) elevating antioxidant levels 75-77. Nevertheless, it is worth mentioning that the cellular response to RT-induced ferroptosis is a highly dynamic and complex process, and many of the cell fate mediators discussed above may present multifaceted functions with both anti-ferroptosis and pro-ferroptosis activities. For instance, RT-induced AMPK activation could induce the phosphorylation of Beclin 1, which could bind to the SLC7A11 module in system xc^-^ to block cystine import and facilitate tumor cell ferroptosis 78. However, energy crisis-induced AMPK upregulation is known to inhibit ACSL4-mediated PUFA synthesis to enhance the ferroptosis resistance of tumor cells 79. Similarly, p53 can promote tumor cell ferroptosis by inhibiting SCL7A11 expression and upregulating arachidonate 15-lipoxygenase expression when the cellular ROS level is high, but switches to an anti-ferroptosis role by suppressing dipeptidyl-peptidase-4 activity when the cellular ROS stress is at a low level. These insights collectively highlighted that the cell fate regulation under RT-induced ferroptosis stress is a highly dynamic and context-driven process and most of the related details are still not well understood, warranting further studies to elucidate the connections between cell fate propensity and RT conditions. Similar to those non-apoptosis cell death forms, ferroptosis shows striking IR dose dependence with negligible ferroptosis levels under low IR doses but increases significantly under high IR doses, and the underlying mechanisms are manifold 26, 80, 81. On the one hand, the ROS surge under low IR doses is within the redox homeostatic capacity of tumor cells and are thus generally tolerable, which is insufficient for fueling the progressive peroxidation of lipid membranes. On the other hand, high IR doses would not only promote ROS production but also trigger the marked activation of p53 signaling, leading to significant downregulation of SLC7A11 while promoting ACSL4 upregulation, further amplifying the lipid peroxidation susceptibility of tumor cells to promote post-RT ferroptosis. Interestingly, it is also notable that tumor susceptibility to RT-induced ferroptosis effects is also profoundly affected by both their genetic and metabolic heterogeneity. Typically, KRAS-mutated tumor cells tend to present elevated ferroptosis suppressor protein 1 (FSP1) expression due to the activation of the MAPK-NRF2 signaling downstream of KRAS, leading to significantly enhancement in their anti-oxidative capacity for eliminating ferroptosis-associated lipid peroxides 82. Alternatively, mutant p53, a frequent mutation in triple negative breast cancer, can regulate *Mgst3* and *Prdx6* in an NRF2-dependent manner to relieve ferroptosis-associated oxidative stress. Consequently, triple negative breast cancer (TNBC) cells expressing mutant p53 tend to show much higher ferroptosis resistance than those expressing wild-type p53 83. On the other hand, the TME is a complex ecosystem comprising numerous tumor cell subtypes with distinct metabolic traits, where the iron, lipid and redox metabolic heterogeneity can induce marked variations in the ferroptosis susceptibility of tumor cell subpopulations. For instance, the expression level of transferrin receptor 1 is positively correlated with the ferroptosis sensitivity of tumor cells 84, while tumor-intrinsic ferritin level shows an inverse correlation with the susceptibility to ferroptosis inducers 85.

### Competing role of autophagy in RT-treated tumor cells: a cell fate choice with both pro-survival and anti-survival functions

Autophagy is an evolutionarily conserved degradation mechanism with major roles in dictating tumor cell survival and death after radiotherapy 86, 87. Based on previous insights, autophagy could be described as a catabolic process to remove cellular waste, cell debris, damaged biomolecules, cellular components and organelles in response to various extracellular and intracellular stresses that are within tolerable range, of which the onset and execution are regulated by multiple nutrient-sensing pathways including mechanistic target of rapamycin (mTOR), sirtuin 1 (SITR1) and AMPK 88, 89. During a typical autophagic process, various danger signals such as nutrient deprivation and cellular damage would trigger the formation of autophagosomes through the interplay with AuTophaGy-related (ATG) proteins, which will then sequester the cellular waste, long-lived biomolecules and impaired organelles and further fuse with the lysosomes for hydrolase-dependent degradation. On account of these insights, autophagy is a crucial degradation program for ensuring cell homeostasis and survival in the context of tolerable stresses 90, 91. Considering that the tumoricidal effect of radiotherapy predominantly relies on its biomolecule-damaging potential, autophagy could substantially contribute to the tumor radioresistance by repairing the RT-induced cellular damage 92, 93. Indeed, Digomann *et al.* reported that radioresistant head and neck squamous cell carcinoma (HNSCC) cells showed substantial upregulation of ATG5, a central mediator of autophagosome formation, to activate autophagy as a post-IR pro-survival mechanism, while low ATG5 expression is usually linked with better radiotherapeutic responses and superior HNSCC patient survival 94. The authors further demonstrated that combining autophagic inhibition using bafilomycin A_1_ or chloroquine with selective SLC3A2 blocking, a key importer of essential amino acids and upstream negative mediator of mTOR autophagic regulatory pathway, could render HNSCC cells more susceptible to RT-induced apoptosis effects by impairing the DNA damage repair cascade and GSH-associated antioxidant defense. Wang *et al.* reported that SMAD4 loss, a common mutation in pancreatic cancer (over 55% of the total patients), contributes to the radioresistance of pancreatic cancer cells through boosting post-IR autophagy. Specifically, the SMAD4 deficiency in pancreatic cancer cells could facilitate the intracellular accumulation of ROS, which acted as a danger signal to activate the autophagic influx. Contrastingly, treating SMAD4-deficient pancreatic cancer cells with ROS or autophagy inhibitors reversed them to a radiosensitive state and markedly amplified the RT-induced tumor cell apoptosis 95. These studies collectively demonstrated that the RT-induced autophagy in tumor cells could exert cytoprotective effects under certain circumstances and suggested the potential application of autophagy inhibition as a radiosensitization modality.

Nevertheless, it is also worth mentioning that autophagy has complex roles and functions in the post-IR cell fate determination of tumor cells, which may also present anti-tumorigenic properties under certain conditions. Clinical data show that only around 20% of the total tumor cell death after radiotherapy was attributed to RT-induced apoptosis, while autophagy is identified as a major contributor to the RT-induced tumoricidal effects 96. The generic mechanism underlying the autophagy-dependent post-IR tumor inhibition is associated with the cell fate decision through the dynamic interplay between autophagic regulation system and the survival stress. Typically, when the RT-induced tumor cell damage exceeds their self-repair capabilities, the autophagic program would eventually lead to autophagic cell death due to the overwhelming accumulation of autophagosomes. Consequently, enhancing autophagy has also emerged as a potential strategy for amplifying the antitumor efficacy of radiotherapy. Nevertheless, the cell fate determination in tumor cells undergoing RT-induced autophagy is affected by a myriad of factors and the associated regulatory mechanisms are still poorly understood, which severely hampers the pharmacological development in this area. For instance, Ko *et al.* observed that while inhibiting ATG5- and Beclin 1-dependent autophagic programs in human and mouse tumor cell lines significantly enhanced their sensitivity to radiotherapy *in vitro* by promoting tumor cell death and decreasing clonogenic survival, it switched to a pro-survival role for the tumors on *in vivo* models by hampering immune cell infiltration and activation 97. Consequently, more in-depth studies are required to enhance our understanding on the RT-induced autophagic programs in tumor cells by desiccating the correlation between cell fate decisions and radiotherapy parameters, which may not only elucidate the autophagic cell fate regulation system in tumor cells but also facilitate the development and optimization of new radiotherapeutic strategies. Indeed, current insights have already revealed multiple contextual factors that may cause the role switch of autophagy in the post-RT conditions, which involves the RT dosing conditions, tumor cell-intrinsic factors and TME traits. Of note, considering the interplay between RT-induced cellular damage and autophagy-enabled repair, it is generally believed that higher RT doses with fewer fractions are generally favorable for overwhelming the autophagy-dependent tumor cell repair capacity under fixed total doses and thus conducive for promoting autophagic cell death. Similarly, the severity of RT-induced mitochondrial damage in tumor cells is positively correlated with their propensity towards autophagic cell death. Meanwhile, tumor cells expressing wild-type p53 are more prone to undergo autophagic cell death after RT than their p53 mutated counterparts. In addition, TME with higher oxygen levels is often correlated with lower autophagy activity, while reducing nutrient supply in the TME could impair the autophagy-dependent repair efficiency through depriving bioenergy and essential molecular substrates, both of which are conducive for promoting post-IR tumor cell death. Moreover, it is also important to consider the impact of the intrinsic genetic and phenotypical heterogeneity of tumor cells on the variable autophagic responses after radiotherapy. Indeed, there is abundant evidence that the expression levels of key autophagy regulators such as microtubule associated protein 1 light chain 3 beta and sequestosome 1 may vary significantly among different subpopulations within a tumor, resulting in different cellular behaviors in response to autophagy-inducing cues 98. The tumor heterogeneity-driven variations in post-treatment autophagic response may further complicate the therapeutic outcome of autophagy-targeting RT modalities, necessitating a deeper understanding of the autophagy regulation network in the complex and heterogenous TME.

### RT-induced senescence of tumor cells

Senescence is a cell fate decision in response to sub-lethal insults and aging, during which various stress factors, especially DNA damage, trigger permanent cell cycle arrest in proliferating cells at G1 or G2 phases 99, 100. Senescence is generally considered as an anti-tumorigenic cell program in normal cells on account of its capability to (1) shutting down the proliferation of defective cells bearing activated oncogenes or loss of tumor suppressor genes and (2) inducing senescence-associated secretory phenotype (SASP) to enhance the infiltration of immune cells 101-103. RT is known to elicit various forms of DNA damage and therefore capable of inducing tumor cell senescence through activating the p53-p16^INK4a^ senescence program 104. However, clinical data suggest that RT-induced tumor cell senescence has significant pro-tumorigenic functions with major roles in orchestrating treatment resistance, relapse and metastasis through multiple pathways. Typically, considering that the regulatory networks of senescence and stemness are largely overlapped with shared mediators including p21, p53 and p16^INK4a^, the RT-activated senescence program in tumor cells would also substantially enhance their stemness features in a cell-autonomous manner, leading to significant enhancement in the self-renewal and invasive capability to facilitate post-treatment relapse 105. Park *et al.* reported that the activation of p16^INK4A^ senescence programs in colorectal tumor cells induced their partial epithelial-mesenchymal transition through enhancing the expression of matrix metalloproteinase-7, contributing to significant increases in the invasion and lymphatic metastasis capacities of the colorectal cancer cells 106.

In addition to these phenotypical and histological alterations, RT-induced tumor cell senescence also exerts significant impact on the composition and functioning of immune systems in the TME. However, reports thus far on the immunoregulatory effects of radiotherapy-induced tumor cell senescence are still debatable. On one hand, multiple studies suggest that RT-induced tumor cell senescence would contribute to the immunosuppression in the TME to facilitate tumor cell survival while preventing post-IR immunostimulatory effects. Indeed, RT would cause the accumulation of senescent cells in TME, leading to the secretion of abundant SASP factors including interleukin 6 (IL-6) and tumor necrosis factor-α (TNF-α). The presence of excessive pro-inflammatory SASP factors in TME would orchestrate chronic inflammation that is detrimental for both innate and adaptive antitumor immune responses, which would recruit immunosuppressive cells to the TME while impairing antigen presentation and inducing T cell apoptosis 107-109. On the other hand, there are also reports that therapy-induced senescence would induce vascular remodeling in pancreatic cancer tissues through promoting SASP-dependent secretion of pro-angiogenetic factors including vascular endothelial growth factor (VEGF), platelet-derived growth factors A and B and fibroblast growth factor 2 as well as MMPs including MMP2/3/7/9/10, leading to the formation of robust vascular networks with activated endothelium that facilitate both the chemotherapeutics delivery and T cell homing 110. Overall, future studies are necessary to determine if these pro-tumorigenic and anti-tumorigenic properties of RT-induced tumor cell senescence are contextual or universal. It is also important to note that the propensity of tumor cells towards senescence in response to cellular stress is closely linked to its genetic profiles. Therefore, the heterogenous genetic makeup of tumors under clinical conditions would significantly complicate the senescent status of tumor cells after radiation exposure, leading to marked diversification of the eventual treatment outcome. For instance, tumor cells expressing wild-type p53 show high propensity towards senescence after radiotherapy, which may contribute to the RT-induced tumor inhibition efficacy 111. Contrastingly, p53-mutant or p53-loss tumor cells are more prone to undergo other cell death modes such as apoptosis or even escape the RT-induced cell cycle arrest for continuous dividing and growth, thus increasing the risk of treatment failure 112. Overall, these reports are in line with the contextual-dependent role of p53 in tumor progression and treatment responses, highlighting the critical influence of tumor heterogeneity in dictating the radiotherapeutic efficacy. As discussed above, the RT-induced tumor cell senescence emerges as a double-edged sword for tumor inhibition. While the induction of tumor cell senescence could temporarily stop tumor growth and contribute to the post-RT tumor inhibition efficacy in the short-term, the accumulation of pro-tumorigenic SASP components such as growth factors (VEGF, hepatocyte growth factor and PDGF), matrix metalloproteinases (MMP-1, MMP-2, MMP-3, MMP-9 and MMP-10) and immunosuppressive cytokines/chemokines as well as inactivation of p53-p21 signaling would allow some tumor cells to escape the cytostatic state while acquiring stem cell-like phenotypes, leading to rapid repopulation of the RT-treated tumors with even higher radioresistance. Consequently, to overcome the senescence associated radiotherapeutic barriers, it is of clinical interest to combine RT with therapeutic modalities inhibiting specific pro-tumorigenic SASP factors by regulating their upstream (NF-κB, Janus kinase/signal transducer and activator of transcription, mTOR, etc) to alleviate their negative impact on the radiotherapeutic outcome in the long-term 113, 114. Meanwhile, considering the pivotal role of p53 in post-RT senescence responses as well as its frequent mutation in various tumor conditions, it is important to develop personalized radiosensitization approaches by taking into the p53 mutation status into account, which should enhance the p53 signaling in tumor cells expressing wild-type p53 to promote the beneficial aspects of RT-induced tumor cells senescence while blocking p53-independent cell fate regulatory pathways in tumor cells expressing mutated p53 to achieve cooperative therapeutic benefit.

### Crosstalk of various post-RT cell fate decisions and the therapeutic implications

RT elicits a myriad of molecular changes in both tumor cells and TME, which direct tumor cells towards various cell fate decisions while also activating pro-survival responses. Typically, considering the complexity of tumor-radiation interaction as well as the interconnection among different cell fate regulatory pathways, the post-RT cell fate determination is not a simple linear process towards specific decisions but often a mixture of cellular outcomes, and their crosstalk is increasingly recognized as a crucial factor on the robustness of RT efficacy and safety. For instance, the adaptive activation of autophagy in breast cancer cell after radiotherapy could direct them towards a senescent phenotype, thus enhancing the secretion of harmful SASP components to promote tumor proliferation and regeneration. Inhibiting autophagy could switch senescence towards apoptosis, which may not only enhance the direct tumor inhibitory efficacy but also abolish the deleterious SASP-dependent bystander effect to reduce the risk of post-RT relapse 115. Alternatively, caspase-8 is widely recognized as a key molecular switch between apoptosis and necroptosis in post-RT tumor cells, which is capable of promoting apoptosis while inhibiting RIPK-dependent necroptosis programs 116. Consequently, deactivating caspase-8 could significantly enhance tumor cell necroptosis after radiotherapy, offering a potential approach for in-situ vaccination through boosting tumor cell immunogenicity and antigenicity. Furthermore, the RT-induced activation of caspase 3 could not only enhance tumor cell apoptosis but also cleave GSDME to activate the pyroptosis programs 117. It is also of interest to note that the RT-induced surge of ROS stress could not only induce mitochondrial dysfunction to trigger cytochrome c-dependent apoptosis pathways but also trigger the iron-catalyzed peroxidation of membrane lipids to drive ferroptosis. Overall, the dynamic and interconnected cell fate regulation after RT could substantially modify the eventual treatment outcome, which may provide promising approaches for improving the efficacy of RT in the clinical context. Indeed, shifting tumor cell fate decision from apoptosis towards other cell death programs may overcome the intrinsic apoptosis resistance of tumor cells and thus contribute to the overall inhibition efficacy. Furthermore, promoting post-RT ICD such as necroptosis, pyroptosis and ferroptosis may elicit local and systemic antitumor immune responses, further reinforcing the durability of the RT-mediated tumor inhibition effects while reducing the risk of tumor metastasis. Nevertheless, considering the strong pro-inflammatory potential of ICDs, it would be necessary to intricately control the apoptosis/ICD ratio of tumor cells after RT to ensure adequate initiation of robust antitumor immune responses while preventing excessive stimulation to induce fatal systemic hyperinflammation, warranting the development of radiotherapeutic modalities with higher controllability.

## Manipulating cell fate for enhancing RT-mediated tumor cell elimination

The insights above collectively demonstrate that modulating the post-IR cell fate decisions holds immense potential for improving the antitumor efficacy of radiotherapy and the therapeutic development in this area is greatly benefited from the advances in synthetic medicinal chemistry. From an overall perspective, these emerging technologies offer novel approaches to alter tumor cell activities after RT to lead them towards or avoid a particular fate, which may not only amplify the direct cellular damage of RT to tumor cells but also harness its immunostimulatory potential to evoke systemic antitumor immune responses for long-lasting protection against tumor relapse and metastasis. Herein, we discuss the general approaches to regulate post-IR tumor cell fate for enhancing the sensitivity of tumors to radiotherapy. Considering the interwoven regulatory networks of various cell fate decisions, the discussions in this chapter are outlined according to the targeted cellular programs (Table 2).

### Enhancing radiosensitivity by abolishing resistance to RT-induced PCD

#### Disrupting cellular redox homeostasis

ROS are highly bioactive substances that may not only evoke direct cellular damage but are also highly involved in multiple cell fate regulatory systems as a signaling factor, presenting a major role in the RT-induced tumor cell death 118, 119. It is understood that the antioxidant systems in tumor cells are crucial for maintaining cellular homeostasis in the context of RT-induced ROS insults and escaping PCD. Consequently, synthetic agents that are capable of promoting redox dyshomeostasis have emerged as promising modalities for tumor radiosensitization by priming them for ROS-mediated PCD cascades. To achieve this purpose, several strategies have been developed and tested on preclinical models, including (1) depleting cellular antioxidants, (2) impairing the expression and functions of antioxidative enzymes and (3) introduction of catalytic species to amplify the bioreactivity and cytotoxicity of ROS generated through RT-induced water radiolysis.

**Figure 2:**
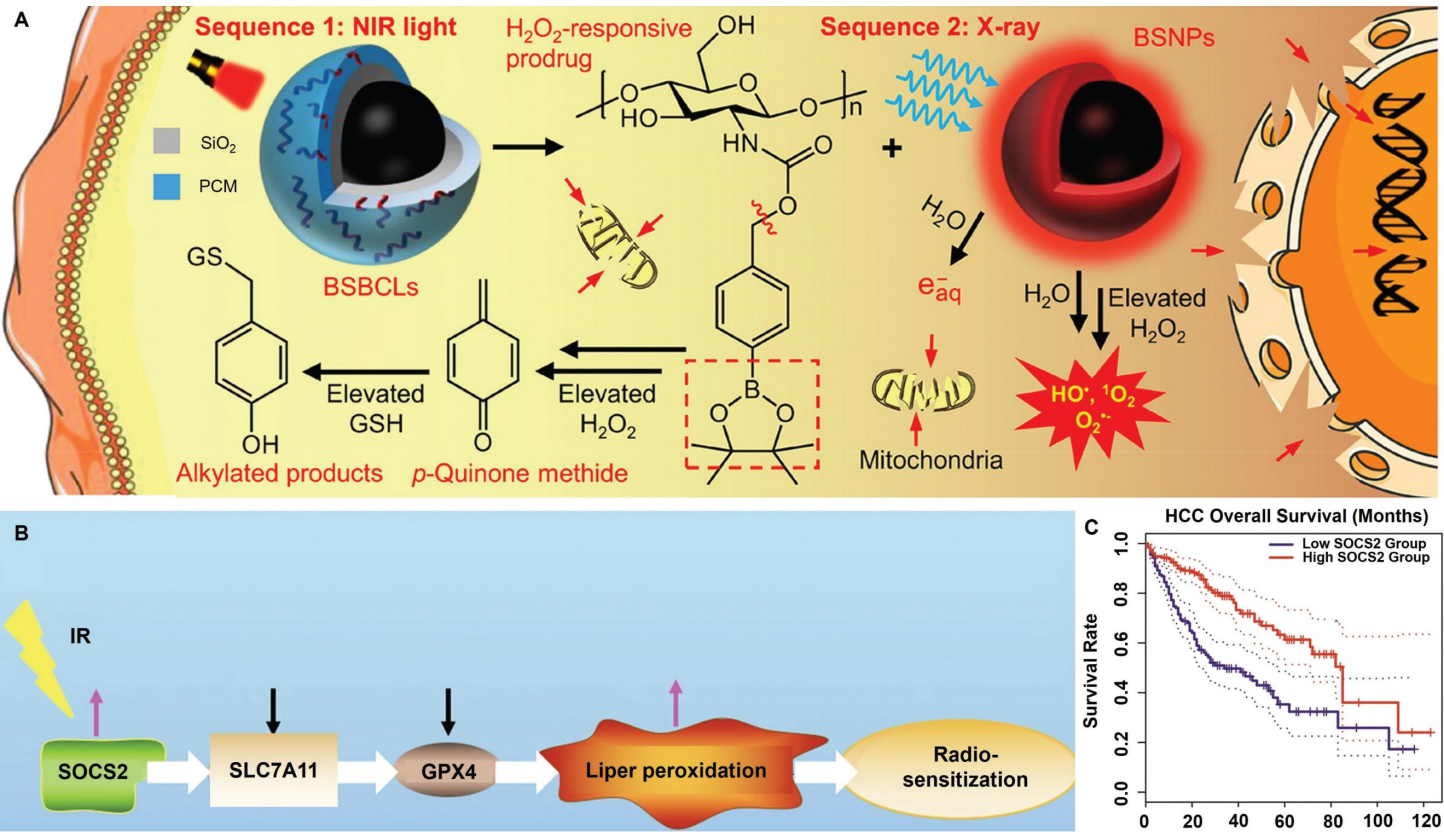
(**A**) Radiosensitization mechanisms for the BiNP-mediated scavenging of tumor-intrinsic GSH for driving post-radiotherapy tumor cell apoptosis. Reproduced with permission from Ref 121. Copyright © 2021 American Chemical Society. (**B**) Molecular pathway of SOCS2-dependent radiosensitization activity. The RT-induced surge of SOCS2 markedly inhibits SLC7A11 to promote ferroptosis of HCC cells after RT. (**C**) Correlation between the SOCS2 expression status and the survival of HCC patients according to TCGA and GEPIA databases, showing the potential role of SOCS2 for HCC radiosensitization. Reproduced with permission from Ref 123. Copyright © The authors.

Tumor cells are known to undergo boosted biosynthesis of various antioxidants including GSH, nicotinamide adenine dinucleotide phosphate (NADPH) and thioredoxin as a compensatory mechanism to counter RT-induced ROS stress. Rashmi *et al.* reported that the drug combination of 2-deoxyglucose (glycolysis inhibitor), buthionine-sulfoximine (GSH inhibitor) and auranofin (thioredoxin inhibitor) substantially enhanced the apoptosis of radioresistant cervix cancer cells after radiation exposure 120. The combinational treatment induced significant elevation of cellular ROS stress after radiotherapy while blocking the tricarboxylic acid cycle activity, leading to the marked activation of pro-apoptotic AMPK pathways as well as p53- and JNK-mediated cell death programs, leading to pronounced inhibition of radioresistant cervix cancers *in vivo* through evoking apoptotic and autophagic cell death. Specifically, even a single dose of only 2 Gy X-ray retarded cervix cancer growth on mouse models by more than 75% compared with the untreated control. Xiang *et al.* conjugated 4-(hydroxymethyl)phenylboronic acid pinacol ester onto chitosan substrates through carbamate ligation to synthesize an ROS-responsive biocompatible prodrug, for which the RT-induced ROS stress can cleave the sp2 C-B bond in the prodrug to release *p*-quinone methide, which could induce the alkylation of GSH to deactivate their ROS scavenging capability. The boronic acid ester-based prodrug effectively induces permanent GSH deletion in tumor cells that led to marked increase in the post-IR apoptosis levels (Figure 2A) 121. Yang *et al.* reported that inhibiting F-box and WD repeat domain containing 7 (FBXW7) in glioma cells could enhance the expression of wild-type and mutant isocitrate dehydrogenase 1 (IDH1) through stabilizing sterol regulatory element binding protein 1 (SREBP1), which leads to significant enhancement in the NADPH-consuming 2-hydroxyglutarate synthesis activities and thus deplete NADPH pool in glioma cells, eventually disrupting the redox homeostasis and sensitizing glioma cells for radiotherapy-induced apoptosis 122. Chen *et al.* reported that suppressor of cytokine signaling 2 (SOCS2) can mediate the ubiquitylation-dependent proteolysis of SCL7A11 in hepatocellular carcinoma (HCC) cells and presents positive correlation with their ferroptosis sensitivity (Figure 2B-C). Inhibiting SOCS2 function in HCC cells effectively impairs GSH biosynthesis and predisposes them for ferroptosis after RT 123. These studies provide a promising rationale of sensitizing tumor cells to radiotherapy by depleting key cellular redox stabilizers.

From a biochemical perspective, the rate and efficacy of antioxidant-mediated ROS scavenging are profoundly affected by the catalytic functions of associated antioxidative enzymes, which present crucial roles in both the ROS detoxification reactions as well as the recycling of exhausted substrates. Consequently, deactivating or deleting key enzymes in major antioxidant systems in RT-treated tumor cells could also contribute to the redox dyshomeostasis to shift tumor cell fate towards various forms of PCD. Ye *et al.* reported that treating HT-1080 fibrosarcoma cells with the combination of RSL3 (GPX4 inhibitor) and Cs-137γ radiation evoked marked ferroptosis while the activation of caspase-dependent cell death programs and DNA damage was only marginal, indicating that blocking GPX4 activity in tumor cells using synthetic inhibitors could synergize with the RT-induced pro-lipoperoxidation effects and creates vulnerability to ferroptosis for high-Z metal free radiosensitization 81. Koppula *et al.* identified that the anti-ferroptosis FSP1-Coenzyme Q10 axis is a major contributor to the radioresistance in KEAP1-inactivated lung cancer cells, a common lung cancer subtype with high risk of relapse and metastasis. Deactivating FSP1 with a synthetic inhibitor iFSP1 markedly enhanced the post-IR lipid peroxidation in several KEAP1-mutant lung cancer cell lines and potentiated efficient lung cancer elimination 124. It should also be noted that most of the studies in this area focus on the ferroptosis-associated antioxidant systems, while reports that exploit other major antioxidant enzymes such as superoxide dismutase and catalase for radiosensitization are still rare, possibly due to the lack of enzyme-specific inhibitors that may impair the radiosensitizing performance while enhancing adverse effects.

**Figure 3:**
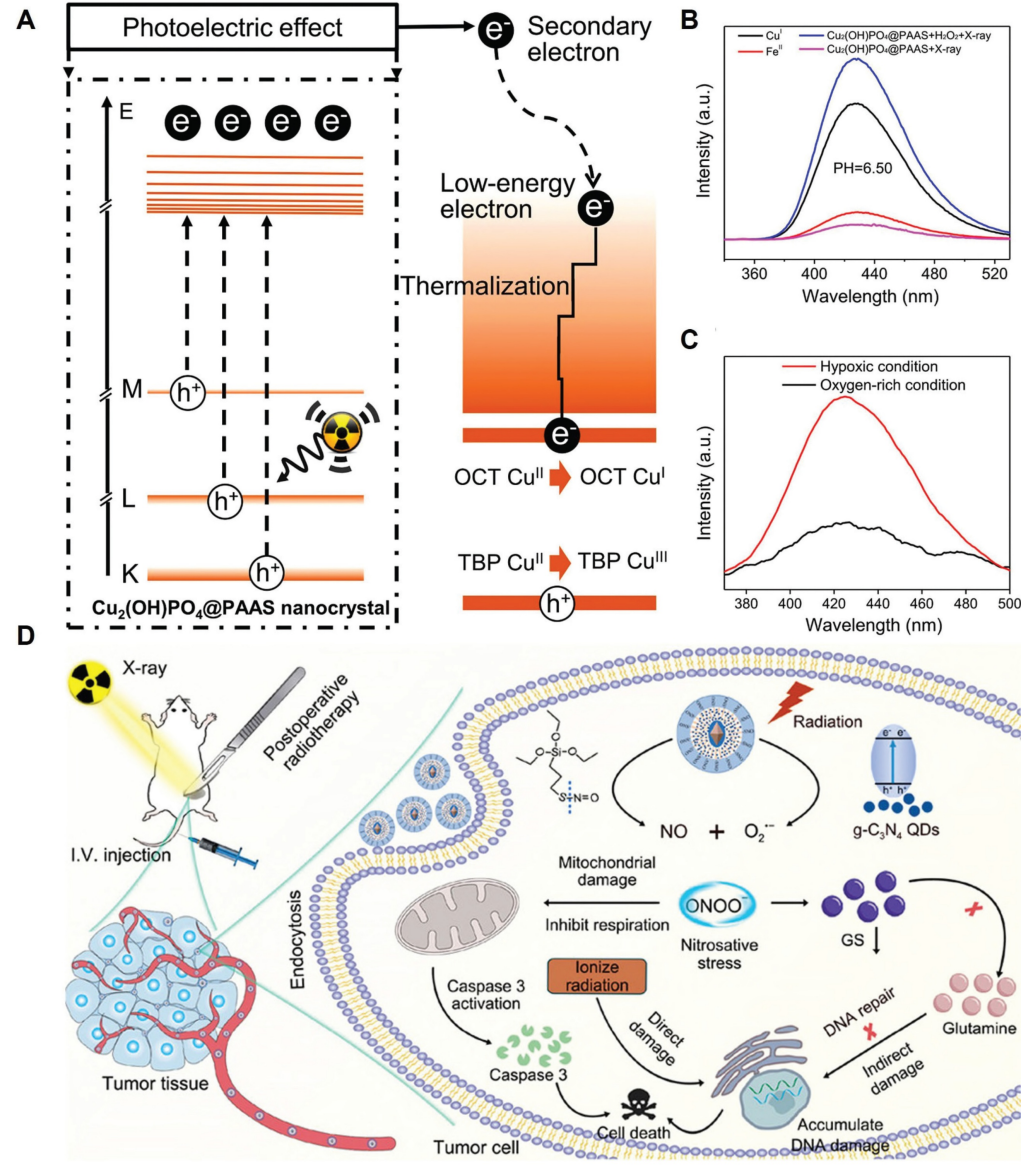
(**A**) Schematic diagram for the X-ray-triggered formation of catalytically active Cu sites for amplifying post-IR ROS stress. (**B**) Fluorescence analysis on the treatment-induced generation of hydroxyl radicals under different conditions. (**C**) Evaluation on the oxygen dependent of the Fenton-like catalytic reactivity of the NP system. The experimental data supported the enhanced hydroxyl radical producing capability of the nanocatalysts under TME-like conditions to drive tumor cell ferroptosis. Reproduced with permission from Ref 127, 2019. Copyright © 2019 American Chemical Society. (**D**) Nanoscintillator-mediated generation of O_2_^•-^ in tumor cells under X-ray excitation and the sequential conversion into peroxynitrite for promoting RT-induced tumor cell death. Reproduced with permission from Ref 129. Copyright © 2022 American Chemical Society.

The RT-induced ROS mostly include superoxide anions (O_2_•^-^) and H_2_O_2_, and their moderate oxidizing potential is becoming increasingly recognized as a limiting factor on the RT-induced tumor cell damage. Hydroxyl radicals (•OH) is one of the most potent oxidizing radicals in biological systems, which is capable of reacting with neighboring biomolecules in an unselective manner including lipids, proteins and nucleotides 125, 126. Consequently, converting RT-induced ROS into hydroxyl radicals using biocatalytic systems appears a promising strategy to enforce various PCD cascades and amplify the tumoricidal potential of radiotherapy. Indeed, several catalytic routes have been proposed to enable the on-demand in-situ conversion of low-toxicity ROS into hydroxyl radicals in tumor cells after radiotherapy. One of the most well-characterized examples in this area is the Fenton/Fenton-like reaction, for which H_2_O_2_ reacts with biocompatible metal ions such as Fe^2+^, Mn^2+^ and Cu^2+^ to produce hydroxyl radical. There is abundant evidence that nanostructures doped with these Fenton catalysts could convert intrinsic and RT-induced H_2_O_2_ in tumor cells into hydroxyl radical to amplify the oxidative stress, thus driving them towards apoptotic or ferroptotic cell death (Figure 3A-C) 127, 128. Alternatively, Liu *et al.* exploited the intrinsic capability of RT to induce O_2_•^-^ generation and developed a silica-based nanoscintillator system by coating an upconverting nanoparticle core with mesoporous silica shells for encapsulating nitric oxide donors and g-C_3_N_4_ quantum dots. The upconverting core could convert part of the incident X-ray into UV light to trigger O_2_•^-^ release, which would undergo a diffusion-limited reaction to produce highly reactive peroxynitrite, eventually leading to marked nitration of tyrosine in intracellular biomolecules (Figure 3D) 129.

#### Impairing DNA repair activities

**Figure 4:**
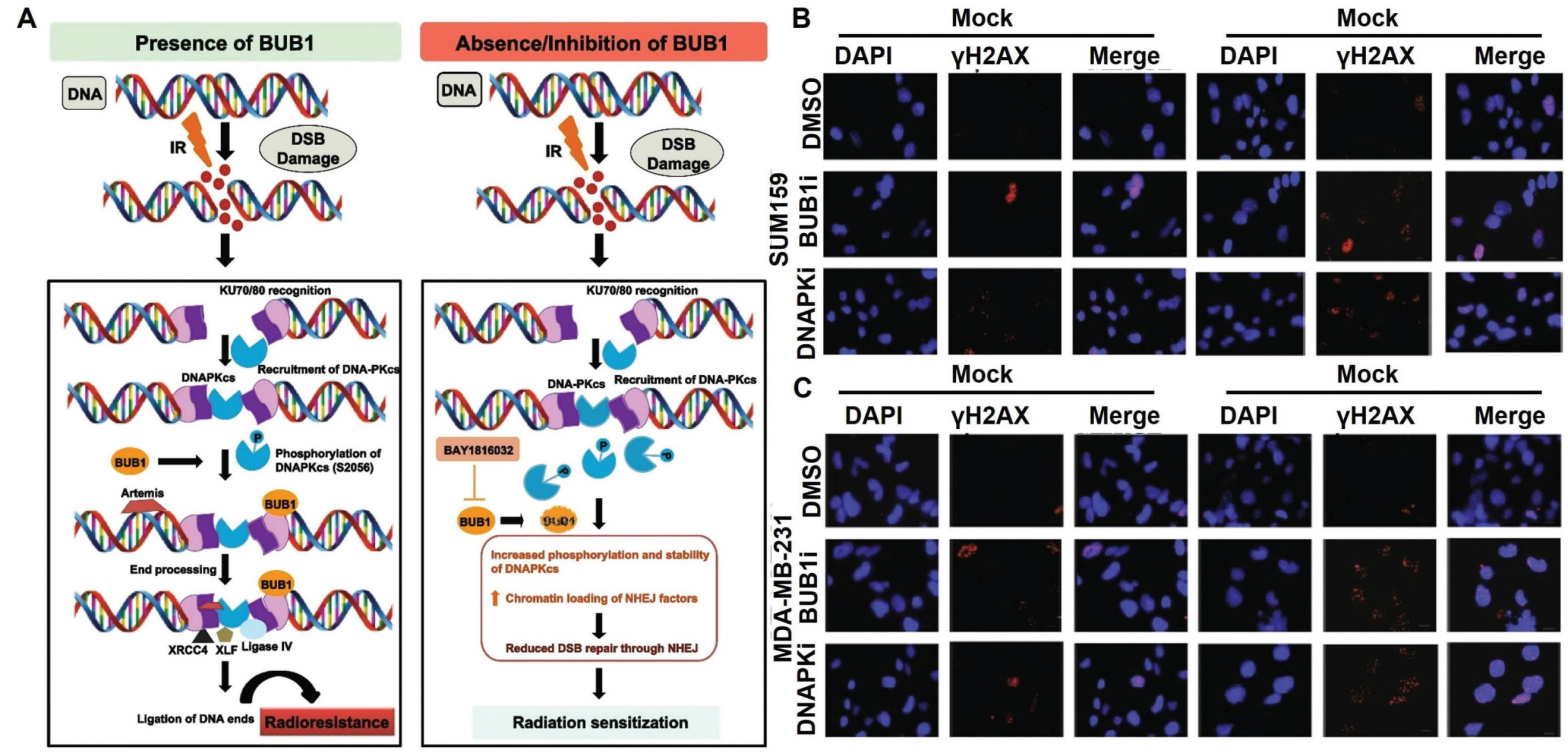
(**A**) NHEJ-inhibition mechanism of BAY1816032 for radiosensitization. (**B-C**) BAY1816032 significantly retards RT-induced NDA repair in SUM159 and MDA-MB-231 cells, thus directing tumor cell fate decision towards apoptosis. Reproduced with permission from Ref 133. Copyright © The Authors.

RT is known to induce various forms of DNA lesions, and failures to repair these lesions would lead to severe consequence including genomic instability and PCD. Double strand break (DSB) is one of the most lethal forms of RT-induced DNA damage, which could activate the DNA damage response in tumor cells to maintain their genomic integrity by harnessing intrinsic DSB repair activities including homologous recombination (HR) and nonhomologous end joining (NHEJ) 130, 131. It is thus anticipated that deactivating key effectors in these two processes could counteract the post-IR DNA repair activities in tumor cells and prime them for PCD. Tsai *et al.* reported that inhibiting histone deacetylase 4 (HDAC4) using synthetic pan-HDAC inhibitor panobinostat or short hairpin RNAs could inhibit the HR activity in RT-treated HCC cells via impairing the DNA repair function of Rad51 to incur persistent DNA damage, leading to pronounced synthetic lethality that significantly promoted apoptosis of HCC cells after radiotherapy 132. Sriramulu *et al.* reported that RT could stabilize the cell cycle Ser/Thr kinase BUB1 in TNBC cells, which would recruit NHEJ proteins to the DSB sites and enhance the phosphorylation of DNA-dependent protein kinase catalytic subunit, thus promoting the NHEJ-dependent DNA repair in TNBC cells to escape apoptosis. Combining radiotherapy with an experimental BUB1 inhibitor BAY1816032 caused significant retardation of the NHEJ-dependent DNA repair efficiency and substantially enhanced the apoptotic rate of TNBC cells, thus prolonging the metastasis survival time of TNBC-bearing mouse models beyond the 60-day observation period (Figure 4) 133. In addition, several inhibitors targeting other DNA damage response mediators are developed and tested in pre-clinical and clinical trials as a neoadjuvant treatment for radiotherapy, of which the notable examples include AZD1390 (inhibitor of ataxia-telangiectasia mutated), AZD6738 (inhibitor of ataxia-telangiectasia and Rad3-related kinases) and AZD2281 (inhibitor of PARP) 134, 135. Notably, in the Phase I trial of AZD1390-sensitized radiotherapy against glioblastoma, AZD1390 was applied administered concurrently with standard-of-care intensity-modulated radiation therapy, which has a maximum tolerated dose (MTD) of 300 mg per day for newly diagnosed primary glioblastoma and 400 mg per day for recurrent glioblastoma (NCT03423628). According to the safety analysis, AZD1390 showed good biocompatibility on real-life patients with no fatal side effects, supporting its further investigation regarding its radiosensitizing benefits.

On the other hand, the ATR inhibitor AZD6738 is typically given through intermittent oral dosing with stand-of-care RT schedules in the Phase I trials for HNSCC treatment, which has an MTD of 240 mg per day for 14 days (NCT02546491, NCT03334617 and NCT02525768). Common toxic side effects of AZD6738 include adverse hematological and gastrointestinal events, although the side effects were considered well tolerable within the MTD. The clinical data revealed a manageable dosing regimen and acceptable safety profile for AZD6738 and supported its further application as a radiosensitizer. As for the PARP inhibitor AZD2281, it was generally given through the oral route when combined with RT for treating glioblastoma (Phase I, NCT01390571), prostate cancer (Phase II, NCT01940188) and pancreatic cancer (Phase I, NCT01908478). The MTD of AZD6738 is largely determined by the cancer indication and RT conditions, which was 100mg once per day with standard RT for glioblastoma treatment, 150 mg twice daily for 14 days when combined with moderately hypofractionated RT for treating prostate cancer and 100 mg twice daily when combined with SBRT for treating pancreatic cancer. Patients receiving AZD2281 showed frequent hematological adverse events including anemia, lymphopenia and neutropenia as a result of its PARP inhibiting function. Meanwhile, AZD2281 also significantly aggravated the RT-associated toxicities such as mucositis and gastrointestinal events, which are usually dose-limiting and generally manageable within the MTD. These insights immediately supported the translational potential of DNA repair inhibitors for disrupting tumor DNA damage responses and promoting post-IR apoptosis.

#### Regulating post-IR cell cycles

**Figure 5:**
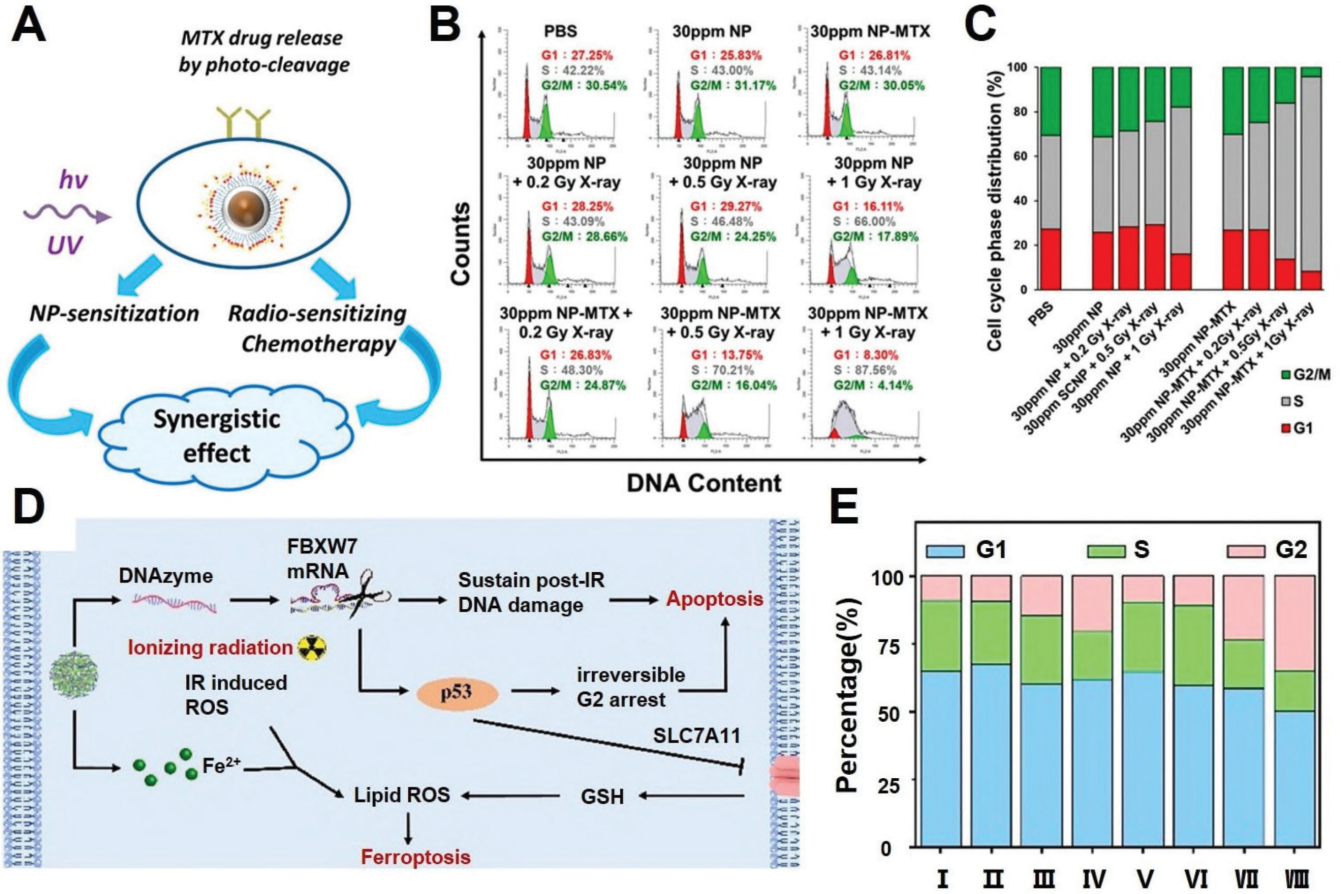
(**A**) Therapeutic mechanism for the NP-mediated synergistic radiosensitization through combining LiYF_4_:Ce^3+^ NPs and MTX. (**B-C**) Cell cycle distribution of pancreatic cancer cells after combined NP and RT, supporting the treatment-induced tumor cycle arrest at the S phase. Reproduced with permission from Ref 136. Copyright © 2021 American Chemical Society. (**D**) Schematic illustration of nanoassembly-enhanced radio-ferroptosis therapy the leveraging cell cycle progression. (**E**) Cell cycle distribution after various treatment, indicating successful post-RT G2 arrest of tumor cells after DNAzyme-mediated deletion of FBXW7 mRNA. Reproduced with permission from Ref 137. © 2023 Acta Materialia Inc. Published by Elsevier Ltd. All rights reserved.

As already described in previous sections, the post-IR cell cycle arrest is a crucial factor for the spatial coordination of various pro-survival programs such as DNA repair, autophagy and cellular homeostasis. Recent insights reveal that acceleration and deceleration of tumor cell cycles after RT could both promote PCD to enhance the radiotherapeutic efficacy. Manoharan *et al.* reported a folic acid-modified nanoparticulate platform for the targeted delivery of methotrexate of pancreatic cancer cells (Figure 5A-C). Notably, the methotrexate contents allow the RT-treated tumor cells to cross the G1-S checkpoint and induce cell cycle arrest at S phase, during which the tumor cells enter a nucleotide depleted state and thus are incapable of enacting the DNA repair programs, eventually leading to a significant enhancement in RT-induced tumor cell apoptosis 136. Alternatively, based on the insight that extended cell cycle arrest at the G2 phase would switch the p53-mediated cell cycle response from pro-survival to pro-apoptosis, our group developed a coordination nanoassembly of ferrous ions and DNAzymes for the targeted degradation of F-box and WD repeat domain containing 7 (FBXW7) mRNA in breast cancer cells, which is an upstream negative regulator of phosphorylated p53 (Figure 5D-E) 137. The nanoassembly-mediated FBXW7 DNAzyme delivery could efficiently degrade tumor-intrinsic FBXW7 to stabilize phosphorylated p53 and induce irreversible G2 arrest to promote tumor cell apoptosis. Meanwhile, the co-loaded ferrous ions could induce iron overload in tumor cells and synergize with radiotherapy to trigger ferroptosis, leading to combinational apoptosis-ferroptosis therapy for enhanced radiosensitization. Overall, the studies above collectively confirmed that post-IR cell cycle progression could be tailored with synthetic agents to disrupt the self-repair programs and enhance their propensity to irreversibly activate the apoptosis sequence.

#### Direct regulation of effector molecules for enacting specific cell fate programs

In addition to the indirect tailoring of the cell fate regulatory network, it is also possible to directly target the effector molecules of certain cell fate programs to enforce or evade the corresponding decisions. A major advantage of this strategy is that it could bypass the defective cell fate regulatory networks and thus overcome the potential PCD resistance. For instance, Yazal *et al.* developed a synthetic autophagy inhibitor EAD1 and confirmed its radiosensitizing effect on pancreatic ductal adenocarcinoma (PDAC) bearing KRAS mutations through blockading post-IR autophagy. The as-developed EAD1 is a synthetic hydroxychloroquine analogue that could impair the fusion between autophagosomes and lysosomes through alkalinization of the acidic lysosomal environment, thus inhibiting the cytoprotective post-IR autophagic influx to divert PDAC cells towards apoptosis. Interestingly, the EAD1-mediated autophagy inhibition showed good inhibitory efficacy against PDAC stem cells on account of their predominant reliance of cytoprotective autophagy program for resisting RT-induced cytotoxic effects, leading to marked reductions in their proliferation and self-renewal capabilities 138. Alternatively, there are multiple studies that DNA methylation-suppressing epigenetic drugs such as decitabine and epigallocatechin-3-gallate could reverse the hypermethylation status of the promotor region for GSDME genes, thus abolishing the tumorigenesis-induced transcriptional inhibition of GSDME expression 139, 140. The treatment induced GSDME upregulation would further synergize with radiation induced caspase activation to promote pyroptosis of tumor cells.

### Enhancing radiosensitivity by promoting post-IR antitumor immune responses

Current insights collectively demonstrate that radiotherapy can not only induce PCD of tumor cells but also modulate the activities of the immune system by altering the immunological traits of TME, offering potential for mounting robust antitumor immune responses to ensure systemic and sustained tumor elimination. Notably, tumor cells are known to escape the recognition and elimination of immune system by (1) eliminating exposure of tumor-associated antigens to reduce immunogenicity and (2) establishing paracrine communication to induce immune cells into immunosuppressive or exhausted phenotypes. From a general perspective, RT could profoundly alter the tumor immune microenvironment and induce both immunostimulatory and immunosuppressive impacts, thus exerting complex influence on the post-RT immune responses 141. On one hand, necrosis/necroptosis, pyroptosis and ferroptosis are generally recognized as highly immunogenic cell death forms, characterized by marked tumor lysis and secretion of various pro-inflammatory factors including DAMPs, cytokine and chemokines, while apoptosis, the dominant cell death form after RT, is generally recognized as an immunosilent cell death program 142. Consequently, the capacity of RT to induce those ICD forms including necroptosis, pyroptosis, ferroptosis largely determines its immunostimulatory potential, which allows the post-RT leakage of danger associated molecular patterns (DAMPs) and tumor-associated antigens for the recognition and processing by tumor-infiltrating immune cells recruited through (1) RT-induced remodeling of aberrant tumor vasculature and extracellular matrix and (2) RT-enhanced secretion of pro-inflammatory chemokines and cytokines, thus activating potent antitumor immune responses 143, 144. Interestingly, it is also worth mentioning that the immunogenic potential of apoptotic tumor cells is strongly affected by the IR doses. After exposure to high IR doses, apoptotic tumor cells may demonstrate plasma-to-membrane translation of CRT as well as enhanced leakage of ATP and HMGB1, all of which are typical features of ICD. On the other hand, RT may also foster an immunosuppressive TME and potentially attenuate the post-RT antitumor immunity. Notably, T cells are commonly recognized as radiosensitive immune cell populations, which would suffer from unneglectable damage after radiation exposure. Meanwhile, radiation exposure may also facilitate the recruitment of immunosuppressor cell populations to the tumor site such as regulatory T cells (Tregs) and myeloid-derived suppressor cells (MDSCs) through secreting specific chemokines (C-C motif chemokine ligand 2, C-C motif chemokine ligand 22, C-X-C motif chemokine ligand 1, etc) and cytokines (transforming growth factor-β, interleukin-10 (IL10), etc), which could potently block T cell activation and inhibit their effector function. Furthermore, RT is known to induce the adaptive upregulation of immune checkpoint programmed death-ligand 1 (PD-L1) on tumor cells, thus promoting tumor immunoevasion and accelerating premature T cell exhaustion. Overall, RT could be a powerful tool for promoting antitumor immune responses for robust and durable tumor inhibition, although its immunosuppressive features should not be neglected for pharmacological development. Although the concept of radio-immunotherapy is still in its infancy, several strategies are already proposed to optimize the immunostimulatory potential of RT, of which the notable examples include (1) application of low-dose RT to reduce collateral immune cell damage and immunosuppressor cell recruitment and (2) cooperation with immune checkpoint inhibition modalities 145-147. In this section, we will discuss the potential application of post-IR cell fate regulation strategies to enhance the radio-immunotherapeutic outcome.

#### Shifting post-IR tumor cell fate towards immunogenic cell death

**Figure 6:**
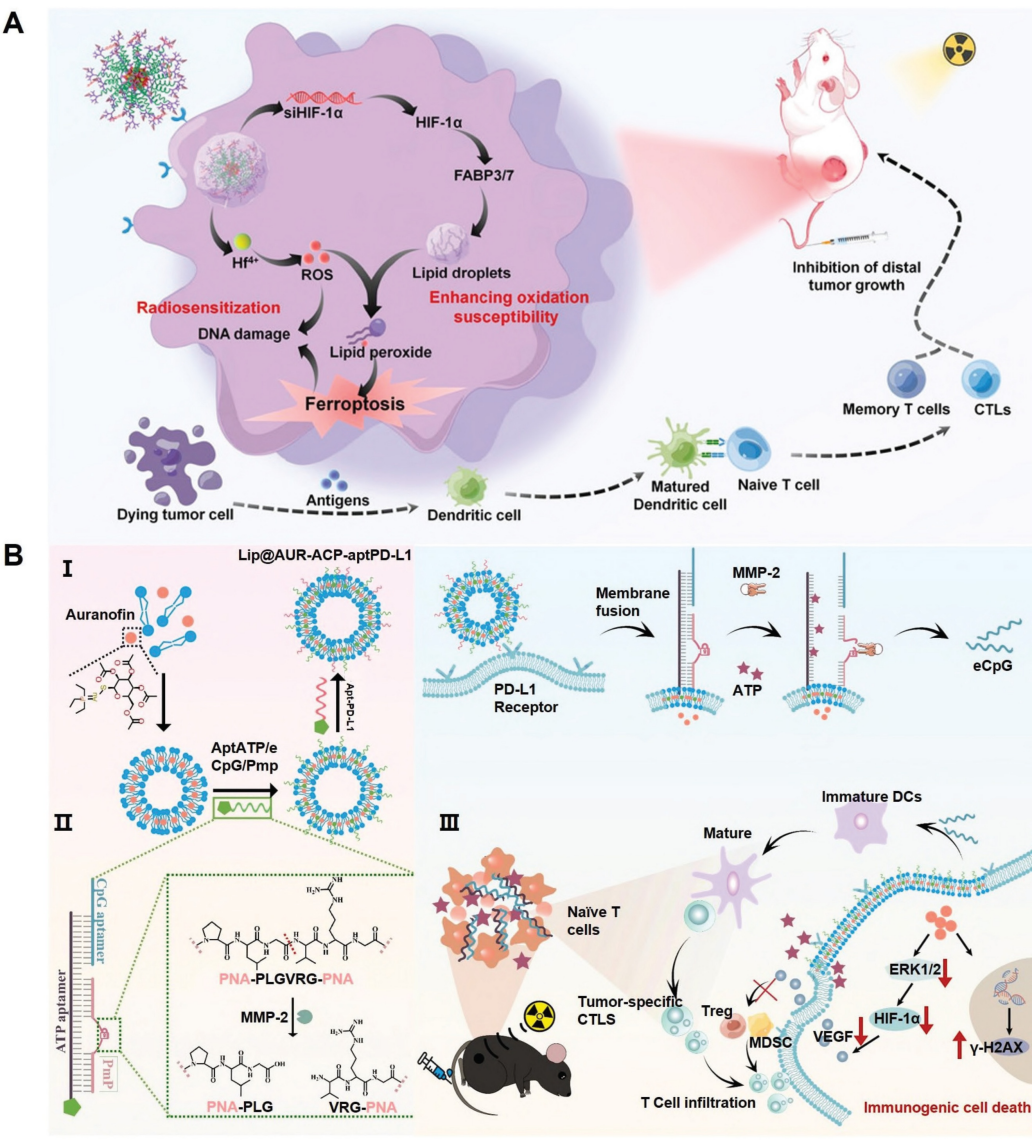
(**A**) Therapeutic mechanisms of the nanoassembly-mediated reprogramming of tumor-intrinsic lipid droplet biogenesis for boosting ferroptosis-enhanced radiotherapy. Blocking the FABP3/7-mediated lipid droplet biogenesis abolishes the detoxification capacity of tumor cells for lipid peroxides, thus aggravating ferroptosis after radiotherapy. Reproduced with permission from Ref 151, 2023. Copyright © 2023 American Chemical Society. (**B**) Construction process of the multifunctional liposomal platform bearing AUR and multivariate-gated aptamer constructs and its activation mechanisms after exposure to low dose radiotherapy, leading to significant enhancement in the post-RT ICD of melanoma cells and DC maturation. Reproduced with permission from Ref 153. Copyright © 2024, The Author(s).

Cell fate is an integral component of the immunoregulation programs under various physiological and pathological conditions. Indeed, several PCD programs including pyroptosis, necrosis, ferroptosis and autophagy have demonstrated potent immunostimulatory capacities featuring leakage of intracellular contents, release of DAMPs and enhanced secretion of immunostimulatory cytokines and chemokines. It is thus anticipated that shifting the fate of RT-treated tumor cells towards immunogenic cell death could synergize with the TME remodeling effect of radiotherapy for mounting robust antitumor immunity. For instance, the cooperation between pyroptosis/ferroptosis and radiotherapy for stimulating antitumor immunity has been implicated in several recent studies on account of their intrinsic biomembrane-disruptive properties, which may significantly enhance the exposure of tumor-associated antigens and DAMPs to stimulate the adaptive antitumor immune responses 148-150. Our group has previously reported that inhibiting lipid droplet biogenesis in tumor cells could amplify post-IR ferroptosis by blocking lipid droplet-mediated elimination of lipid peroxide from damaged tumor biomembranes (Figure 6A) 151. Specifically, the pro-ferroptosis therapeutics was synthesized through complexing Hf^4+^ ions and hypoxia-inducible factor 1α-inhibiting siRNAs onto tumor-targeting polymeric assemblies. After entering tumor cells, the siRNAs could inhibit HIF-1α expression and block the downstream fatty acid transporters fatty acid binding protein (FABP) 3 and FABP7 to deplete the lipid droplet pool in tumor cells. This would significantly amplify the ferroptosis-associated lipid peroxidation in various biomembranes, leading to enhanced fragmentation and disruption in key cellular membrane structures to release the contents for activating the tumor-specific immune responses.

Alternatively, Xu *et al.* developed a maleimide-modified CpG-loaded Fe_3_O_4_ nanoparticle as nanoadjuvants for radiotherapy-triggerable *in situ* tumor vaccination. Treating tumor cells with the iron-based nanoadjuvants would induce iron overload and glutaminolysis to evoke pronounced ferroptosis after RT, leading to marked release of tumor-associated antigens into the TME 152. Notably, the maleimide moieties on nanoadjuvants surface could scavenge the sulfhydryl-rich neoantigens and facilitate their uptake by tumor-infiltrating antigen-presenting cells to enhance the vaccination efficacy. Our group also reported a multifunctional liposomal system by modifying auranofin (AUR)-loaded fusogenic liposomes with multivariate-gated aptamer constructs for enhancing the radio-immunotherapeutic response of melanoma (Figure 6B) 153. Notably, the AUR content could enhance RT deposition in tumor tissues to promote ICD of melanoma cells in the context of low-dose radiotherapy, leading to efficient release of tumor-associated neoantigens and various DAMPs such as ATP. Notably, the AUR-augmented ATP release could synergize with RT-induced upregulation of matrix metalloproteinase-2 to trigger the AND-gate release of engineered cytosine-phosphate-guanine aptamers from the surface-bound aptamer constructs to promote the maturation of tumor-infiltrating DCs for mounting robust T cell-mediated antitumor immunity. Overall, these studies supported the applicability to promote post-IR immunogenic tumor cell death for maximizing the immunostimulatory potential of radiotherapy.

#### Remodeling tumor-immune cell communication

**Figure 7:**
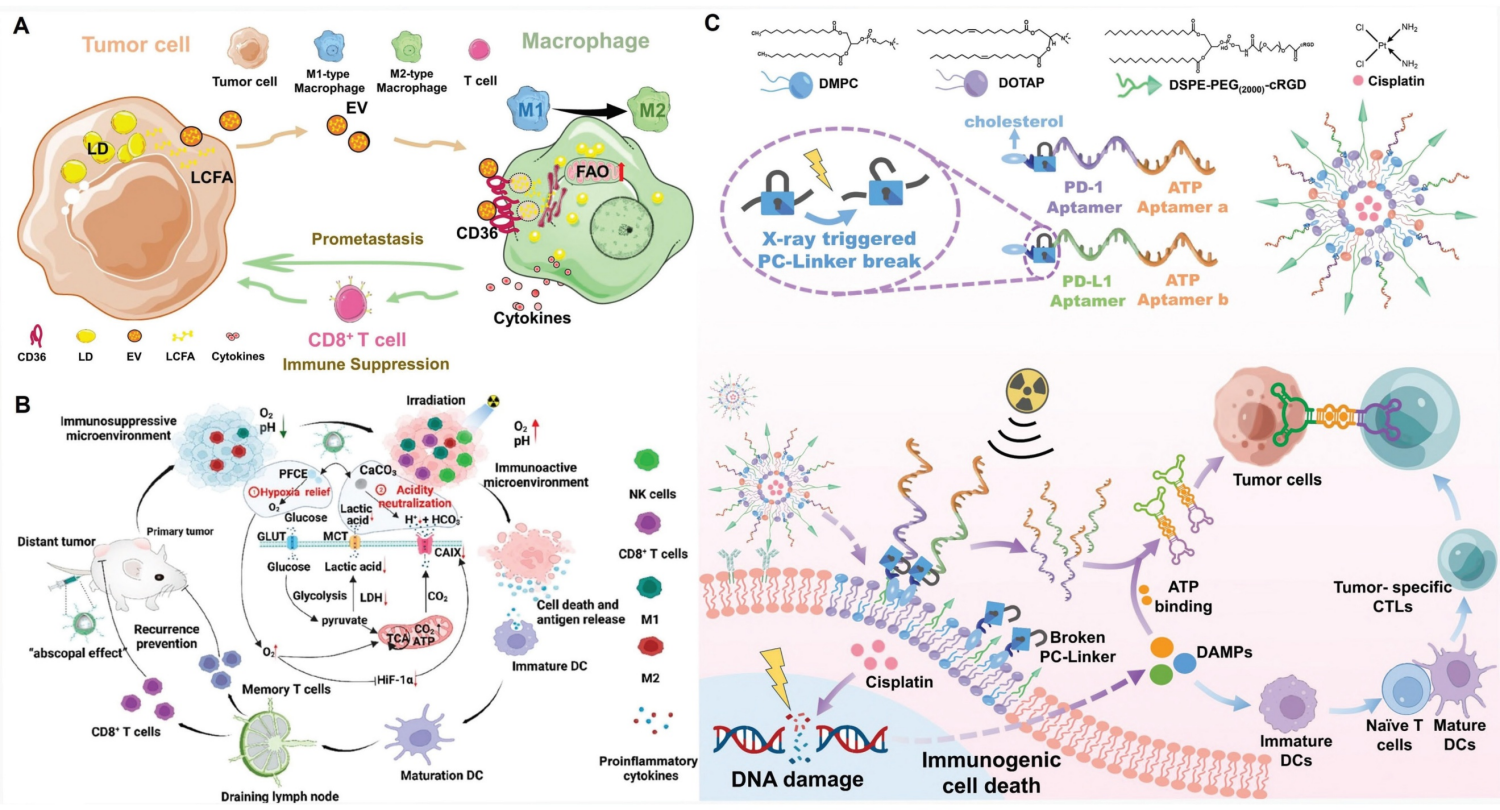
(**A**) Tumor cells secrete lipid-containing vesicles to reprogram tumor-infiltrating macrophages for attenuating CD8+ T cell-mediated antitumor immunity. Reproduced with permission from Ref 154, 2022. Copyright © The authors. (**B**) Synthetic modulation of the lactate-associated glycometabolism in tumor cells for boosting radio-immunotherapy using PFCE@fCaCO_3_-PEG nanoparticles. The PFCE@fCaCO_3_-PEG NPs enabled in-situ oxygen supplementation to enforce the metabolic transition of tumor cells from glycolysis to oxidative phosphorylation, thus attenuating lactate accumulation in TME to reverse the immunosuppressive features. Reproduced with permission from Ref 160. Copyright © 2022 American Chemical Society. (**C**) Construction of the multifunctional nanoradiosensitizers and its radiosensitization and on-demand T cell engaging mechanisms after RT. RT triggers the in-situ formation of aptamer-based PD-L1-PD-1 bispecific T cell engagers in TME to kickstart the cancer-immunity cycles for improving the T cell-mediated antitumor immune responses. Reproduced with permission from Ref 163. © 2024 Elsevier Ltd.

The RT-induced cell fate divergence is not only a determining factor of the tumor-intrinsic immunogenicity, but also has profound impacts on tumor-infiltrating immune cells as a whole through establishing a complex intercellular communication network, which significantly contributes to the attenuation of RT-associated immunostimulatory benefits by (1) impairing antigen presentation, (2) inducing T cell exhaustion and (3) recruiting immunosuppressor cells. Consequently, regulating post-IR tumor cell fate decisions to remodel tumor-immune cell communication could a viable approach to start the cancer immune cycle for eliciting robust radiotherapy-augmented antitumor immunity. For instance, several recent studies suggest that lipid peroxides released by ferroptotic tumor cells could be captured by CD36-expressing immune cell populations including dendritic cells, macrophages and cytotoxic T cells and directly elevate the ROS stress in immune cells, which would cause significant mitochondrial damage while driving the immune cell towards ferroptosis and apoptosis, eventually leading to impaired cytotoxic capacity and premature exhaustion of infiltrating T cells (Figure 7A) 154-156. This phenomenon also challenges current ferroptosis-dependent radio-immunotherapeutic paradigms, which may require the integration of certain lipid peroxide scavenging modalities to minimize the negative impact on immune cells while not affecting the ferroptotic damage to tumor cells. Alternatively, radiotherapy-induced senescent tumor cells universally demonstrate enhanced glycolysis phenotypes due to RT-induced upregulation of key glycolysis enzymes such as lactate dehydrogenase A and pyruvate kinase M2 157-159. The RT-induced lactate accumulation in TME would not only aggravate the microenvironmental acidity to profoundly inhibit the proliferation and cytotoxic activity of tumor infiltrating cytotoxic T cells, but also promotes the immunosuppressive function of lactate-avid immunosuppressor cell populations including Tregs and MSDCs. Specifically, the accumulation of lactic acid in TME could fuel the biological activity of metabolically-flexible Tregs and MSDCs while inhibiting the bioenergy production in glycolysis-avid T cells, leading to a significant increase in the immunosuppressor/effector cell ratio in the post-RT TME. Meanwhile, lactic acid could directly stimulate the immunosuppressive function of both Tregs and MDSCs. Typically, lactic acid could activate Treg-intrinsic FOXP3 signaling to upregulate cytotoxic T-lymphocyte-associated protein 4 expression and IL10 secretion, while also promote the differentiation of monocytes into MSDCs and stimulate their expression levels of immunosuppressive molecules such as Arginase1 and T cell anergy factors. Furthermore, the presence of lactic acid in the TME would also promote the M2-like polarization of tumor-associated macrophages through activating HIF-1α signaling, contributing to the orchestration and maintenance of the immunosuppressive TME. It is thus anticipated that combining radiotherapy with senolytic drugs or lactate depleting treatment TME may alleviate the radiotherapy-associated immunosuppressive effects in the TME and enhance T cell-mediated tumor cell elimination (Figure 7B) 160. Furthermore, considering the potent capability of tumor cells to evade the attack by effector T cells despite radiotherapy-induced immunostimulatory effects, there is increasingly interest to direct T cell-mediated cytotoxicity to tumor cells in the post-RT TME to amplify the radio-immunotherapeutic efficacy 161, 162. For instance, He *et al.* developed an RT-activatable supramolecular nanoradiosensitizer by modifying cisplatin-loaded fusogenic liposomes with molecularly engineered aptamer precursors (Figure 7C) 163. The cisplatin content could promote radiotherapy-induced tumor cell ICD through photoelectric mechanism to facilitate T cell activation, while the aptamer components could further self-assemble into PD-L1-PD-1 bispecific T cell engagers in the TME in an in-situ manner to enable direct binding of PD-1-expressing cytotoxic T cells onto PD-L1-upregulated RT-exposed tumor cells, thus kickstarting the post-RT cancer-immunity cycle while antagonizing PD-L1 immune checkpoint for improving the T cell-mediated antitumor responses. Nevertheless, it should be mentioned that the development and application of bispecific cell engager technology is still in the early stages, warranting further evaluation of their efficacy and safety on clinically relevant models.

## Conclusion and perspectives

The rapid advances in the concepts and technologies of radiotherapy have brought renewed hopes for the clinical intervention against a variety of tumor indications. Development of novel radiosensitization strategies is a topic of both scientific and practical interest, which may not only deepen our understanding on various biochemical aspects of radiation-biointeraction but also improve the efficacy and safety of radiotherapy on real life patients. Remarkably, the post-IR cell fate divergence has demonstrated critical importance in determining the radiotherapeutic efficacy and revealed numerous druggable targets for radiosensitization, holding promise to promote tumor cell death and evoke antitumor immune responses. Indeed, cell fate regulatory therapeutics could either be used alone or in conjunction with other clinically tested high Z metal-based radiosensitizing agents and have demonstrated plausible therapeutic benefits in a myriad of preclinical and clinical studies, supporting their translational potential in a clinical context. Notably, nanoradiosensitizers have demonstrated significant promise to address the existing limitations of radio-immunotherapy in the clinic. Integrating high Z metal ions and cell fate regulatory therapeutics into nanosystems of tailored sizes, shapes and surface characteristics could not only reduce their collateral damage through preventing premature leakage and non-specific uptake, but also facilitate the development of multimodal therapies for achieving novel therapeutic synergisms. Remarkably, the high Z metal-enabled radiosensitizing effects allowed adequate inhibition of the tumors at a relatively lower total radiation doses, which is beneficial for ameliorating radiation damages to healthy cells and tumor-infiltrating immune cells without impairing the antitumor efficacy, thus showing particular relevance for boosting radio-immunotherapeutic responses 164. Interestingly, a plethora of synthesis strategies have been developed for the facilely integration of high Z metal species into functional nanoplatforms including coordination-driven doping and nanoparticle encapsulation technologies, which offer ample opportunities to develop multifunctional nanoradiosensitizers for personalized and effective radio-immunotherapy 165, 166. Currently, two types of high Z metal-integrated nanoparticular radiosensitizers are under active clinical trial for the treatment of multiple tumor indications, which are the Gd-based AGuIX (NCT04789486, NCT03308604, NCT04881032, NCT03818386 and NCT04899908) and Hf-based NBTXR3 (NCT01946867, NCT01433068, NCT04484909, NCT02379845 and NCT02465593) 167. Specifically, AGuIX is developed through chelating Gd ions onto nanoscale polysiloxane substrates, which could be well-tolerated *in vivo* without showing obvious toxicity even under a high dose of 100 mg/kg. Meanwhile, NBTXR3s are essentially crystalline HfO_2_ nanoparticles stabilized by negatively charged phosphate polymers, which have already been approved by FDA and EMA for RT enhancement. Nevertheless, while the integration of high Z metal species into nanotherapeutic systems offers many clinically favorable merits including optimized pharmacokinetics, enhanced tumor-specificity and multifunctionality, the clinical translation of nanomedicine-based radiosensitizers still faces multiple major obstacles, evidenced by the limited number of approved nanoradiosensitizers in the market worldwide. Indeed, nanoengineering of the radiosensitizing components would significantly complicates their interaction with the biological systems at molecular, cellular, tissue and systemic levels, warranting comprehensive evaluation of their radiosensitizing efficacy and safety under clinical conditions in the long term. On the other hand, the increasing complexity in drug design and preparation significantly hinder the upscale production of these nanoradiosensitizers, posing substantial manufacturing, financial and logistic challenges for their broad application in the clinic.

Several challenges are also noted for the clinical application of those cell fate regulatory radiosensitizing therapeutics. Notably, proper functioning of the cell fate determination programs is crucial for ensuring the correct development and homeostasis maintenance of normal tissues, which involve the participation and precise coordination of those cell fate mediators discussed in previous sections. As current cell fate-targeting therapeutics lack intrinsic cell selectivity to differentiate tumor cells and healthy cells, it is understood that the non-discriminative cell fate modulation would induce unacceptable toxicity. Remarkably, the recent advances in drug delivery nanotechnology offer novel approaches to overcome these pharmacokinetic challenges. Specifically, the nanocarrier systems not only potentiate the targeted delivery of the cell fate-targeting therapeutics to tumor cells, but also allow their spatial-temporally controlled release from the carrier substrate, thus enabling even greater synergism with radiotherapy 168, 169.

Meanwhile, it is also notable that there is still a significant discrepancy between the experimental results on mouse models and the therapeutic performance on real life patients. Particularly, the impact of radiotherapy on various cell fate programs seems to be highly context-dependent, and alterations in the RT dose, fractionation and radiation types may induce significant changes in the eventual cell fate decisions. For instance, while low-dose radiotherapy is prone to inducing immunosilent apoptosis in tumor cells, it demonstrates significantly higher capacity to repolarize immunosuppressive tumor-associated macrophages into anti-tumorigenic M1 phenotype and thus more favorable for radio-immunotherapy under certain circumstances 170. Alternatively, Bodo *et al.* reported that a single RT dose of 24 Gy presented higher apoptosis-inducing capacity than conventional fractionated radiotherapy due to its vasculature damaging effect, which could cause reperfusion injury in the irritated site and impair the tumor-intrinsic HR-dependent DNA repair program through triggering SUMO stress 171. However, there are also reports that while a single high RT dose may evoke greater direct damage to tumor cells, the accompanying tumor vasculature collapse would aggravate local hypoxia and impede the infiltration of immune cells into the irritated tumor tissues, thus significantly compromising the post-IR antitumor immune responses 172-174. These insights collectively support the complexity of radiation-biointeraction and necessitate detailed investigation on clinically relevant models.

Furthermore, tumor heterogeneity is a crucial clinic factor dictating the success of RT on real-life patients. In terms of the molecular and phenotypical heterogeneity, the complex genetic makeup of tumor cells in a tumor may undermine the tumoricidal and immunostimulatory effects of RT at varying degrees. For instance, mutations in key cell fate regulators such as p53 and RAS would cause significant changes in the sensitivity of tumor cells to specific cell death decisions, further promoting post-RT tumor cell survival and regeneration. On the other hand, tumors are highly complex ecosystems with marked microenvironmental heterogeneity, which may also contribute to the radioresistance. Typically, the aberrant metabolism and angiogenesis of solid tumors would establish abundant hypoxic regions throughout the tumor tissues, which not only attenuate the ROS-inducing potential of RT to restrict its cytotoxic effects but also harbor those cancer stem cells with enhanced radioresistance and self-renewal capabilities, thus contributing to the post-RT tumor survival and regrowth. Meanwhile, solid tumors frequently present a highly heterogenous immune cell landscape that may substantially influence the recruitment, activation and expansion of anti-tumorigenic immune cells, further influencing the immunostimulatory potential of RT. In summary, the molecular and histological heterogeneity emerges as a formidable barrier for RT through escaping cell death and limiting the immunostimulatory potential, warranting the development of more personalized RT-sensitizing modalities to improve the treatment outcome.

## Figures and Tables

**Table 1:** Summary of typical cell fate regulatory strategies for radiosensitization.

Cell fate decisions	Molecular targets	Therapeutic approaches	Radiosensitizing mechanisms
**Apoptosis**	BCL-2 families, Caspases, p53	Promoting apoptosis	Overcoming intrinsic apoptosis resistance in tumor cells to promote RT-induced tumor cell death
**Necrosis** **/Necroptosis**	RIPK1, RIPK3, MLKL	Promoting necrosis	Enhancing RT-induced tumor cell necrosis/necroptosis to promote antitumor immune responses
**Pyroptosis**	Caspases, Gasdermins, NLRP3	Promoting pyroptosis	Enhancing RT-induced tumor cell pyroptosis to promote antitumor immune responses
**Ferroptosis**	GPX4, SLC7A11, FSP1, ACSL4	Promoting ferroptosis	Enhancing post-IR tumor cell ferroptosis for direct inhibition and immunostimulation
**Autophagy**	mTOR, Beclin-1, ATG5, p62	Blocking autophagy	Inhibiting post-IR self-repair to promote tumor cell death
		Promoting autophagic cell death	Inducing excessive autophagy to drive autophagic tumor cell death
**Senescence**	p53, p16, mTOR, CDK4/6	Leveraging SASP	Abolishing pro-tumorigenic SASP components to prevent regeneration
		Combining senolytic therapy	RT-mediated conditioning of tumor cells into a senescent state for promoting the efficacy of senolytics

**Table 2:** Summary of recent studies on regulating tumor cell fate decisions for improving RT outcome.

Fate type	Targets	Systems	Models	Mechanism	Efficacy	Ref
**Apoptosis**	AMPK, P53, JNK	2-DG, buthionine-sulfoximine and auronofin	Cervix cancer-bearing mouse models	Impairing antioxidative defense, blocking TCA cycle	Inhibition rate >75%	120
**Apoptosis**	GSH	4-(hydroxymethyl) phenylboronic acid pinacol ester-conjugated chitosan nanoparticles	TNBC mouse models	Post-RT depletion of GSH to exhaust antioxidative defense	Inhibition rate ~98%	121
**Apoptosis**	FBXW7	CRIPSR-Cas9 vectors	*In vitro* patient-derived glioma samples	Genetic inhibition of FBXW7	Inhibition rate >97%	122
**Ferroptosis**	SOCS2	Gene vectors	Patient-derived HCC samples and xenografts on mice	Inhibiting SLC7A11 to promote post-RT ferroptosis	Inhibition rate > 50%	123
**Ferroptosis**	GPX4, system xc-	RSL3, IKE	Fibrosarcoma xenograft mouse models	Inhibiting GSH-mediated anti-ferroptosis defense	Complete regression *in vivo*	81
**Ferroptosis**	FSP1	iFSP1	KEAP1-mutant patient-derived lung cancer xenograft on mice	Inhibiting FSP1-CoQ10 axis	Inhibition rate > 80%	124
**Apoptosis/** **necrosis**	Cellular ROS	Cu_2_(OH)PO_4_ nanocrystals	HeLa tumor bearing mice	Converting ROS into hydroxyl radicals	Inhibition rate > 90%	127
**Apoptosis**	Caspase 3	Silica-based nanoscintillators	Colon cancer mouse model	Promoting peroxynitrite generation	Inhibition rate ~90%	129
**Apoptosis**	HDAC4	Panobinostat	HCC xenografts on mice	Blocking Rad51	Inhibition rate ~90%	132
**Apoptosis**	BUB1	BAY1816032	TNBC mouse models	Blocking NHEJ pathways	Inhibition rate > 80%	133
**Apoptosis**	G1-S checkpoint	Methotrexate-loaded folic acid modified nanoparticles	Pancreatic cancer mouse models	Inducing S phase arrest	Inhibition rate > 70%	136
**Apoptosis/ ferroptosis**	FBXW7	HA-modified FBXW7-Fe coordination nanoassemblies	TNBC mouse models	Inducing irreversible G2 arrest, blocking NHEJ activity	Inhibition rate > 95%	137
**Apoptosis**	Autophago-somes	EAD1	PDAC mouse model	Inhibiting autophagy	Inhibition rate > 80%	138
**Pyroptosis**	DNA methyltransferase	Metal-phenolic nanocoordinator	TNBC mouse model	Inhibiting DNMT to restore GSDME expression	Inhibition rate > 60%	139
**Pyroptosis**	Caspase-3	DAC-loaded HfO_2_ NPs	TNBC mouse model	Ehancing caspase 3 and GSDME	Inhibition rate > 85%	140
**Ferroptosis**	FABP3/7	Hf^4+^/siHIF-1α-loaded nanoassemblies	TNBC mouse model	Blocking lipid droplet biogenesis	Inhibition rate ~92%	151
**Ferroptosis**	Iron metabolism	CpG-loaded Fe_3_O_4_ nanoparticles	TNBC mouse model	Inducing iron overload while stimulating DCs	Inhibition rate > 90%	152
**Multimodal ICD**	HIF-1α-VEGF axis	Aptamer-engineered fusogenic liposomes	Melanoma mouse model	VEGF inhibition, AUR-mediated radiosensitization and CpG delivery	Inhibition rate > 90%	153
**Multimodal ICD**	Glycolysis	Fluorinated CaCO_3_ nanoregulator	CT26 and 4T1 mouse model	Reversing TME acidity and hypoxia	Inhibition rate > 95%	160
**Multimodal ICD**	PD-L1	Aptamer-engineered fusogenic liposomes	TNBC mouse model	Post-RT PD-L1-PD-1 bispecific tumor-T cell engagement	Inhibition rate > 95%	163
